# Development of a semi-automated MHC-associated peptide proteomics (MAPPs) method using streptavidin bead-based immunoaffinity capture and nano LC-MS/MS to support immunogenicity risk assessment in drug development

**DOI:** 10.3389/fimmu.2023.1295285

**Published:** 2023-11-10

**Authors:** M. Violet Lee, Ola M. Saad, Sylvia Wong, Jason LaMar, Lynn Kamen, Ben Ordonia, Rachel Melendez, Azadeh Hassanzadeh, Shan Chung, Surinder Kaur

**Affiliations:** Department of Bioanalytical Sciences, Genentech, Inc., South San Francisco, CA, United States

**Keywords:** MHC associated peptide proteomics (MAPPs), immunogenicity risk assessment, *in vitro*/ex vivo, biotherapeutics, major histocompatibility complex class II (MHC II), LC-MS, immunoaffinity capture, streptavidin magnetic beads

## Abstract

Major histocompatibility complex (MHC)-Associated Peptide Proteomics (MAPPs) is an *ex vivo* method used to assess the immunogenicity risk of biotherapeutics. MAPPs can identify potential T-cell epitopes within the biotherapeutic molecule. Using adalimumab treated human monocyte derived dendritic cells (DCs) and a pan anti-HLA-DR antibody (Ab), we systematically automated and optimized biotin/streptavidin (SA)-capture antibody coupling, lysate incubation with capture antibody, as well as the washing and elution steps of a MAPPs method using functionalized magnetic beads and a KingFisher Magnetic Particle processor. Automation of these steps, combined with capturing using biotinylated-Ab/SA magnetic beads rather than covalently bound antibody, improved reproducibility as measured by minimal inter-and intra-day variability, as well as minimal analyst-to-analyst variability. The semi-automated MAPPs workflow improved sensitivity, allowing for a lower number of cells per analysis. The method was assessed using five different biotherapeutics with varying immunogenicity rates ranging from 0.1 to 48% ADA incidence in the clinic. Biotherapeutics with ≥10%immunogenicity incidence consistently presented more peptides (1.8-28 fold) and clusters (10-21 fold) compared to those with <10% immunogenicity incidence. Our semi-automated MAPPs method provided two main advantages over a manual workflow- the robustness and reproducibility affords confidence in the epitopes identified from as few as 5 to 10 donors and the method workflow can be readily adapted to incorporate different capture Abs in addition to anti-HLA-DR. The incorporation of semi-automated MAPPs with biotinylated-Ab/SA bead-based capture in immunogenicity screening strategies allows the generation of more consistent and reliable data, helping to improve immunogenicity prediction capabilities in drug development.

MHC associated peptide proteomics (MAPPs), Immunogenicity risk assessment, *in vitro*/ex vivo, biotherapeutics, Major Histocompatibility Complex Class II (MHC II), LC-MS, Immunoaffinity Capture, streptavidin magnetic beads

## Introduction

Biotherapeutics, such as monoclonal antibodies (mAbs), represent an increasing share of the new drugs that are approved by regulatory agencies (1). Biotherapeutics have unique characteristics that give them an advantage over small molecule drugs, such as longer half-life and greater specificity. However, all therapeutic proteins have the potential to elicit an immune response (2, 3). Immunogenicity can manifest in a host of ways that may affect the safety, efficacy, pharmacokinetics (PK), and/or pharmacodynamics (PD) of a molecule. Efforts to reduce mAb immunogenicity either by reducing differences in protein sequences from antibody variants produced naturally in humans through mAb humanization or by developing fully human mAb have not eliminated immunogenicity concerns (4). With the growing number of biotherapeutics in development, including those already in clinical trials and nearing approval, health authorities are requiring thorough integrated immunogenicity risk assessments as part of IND submission (EMA Immunogenicity Guidance 2017, FDA Immunogenicity Draft Guidance 2022). Therefore, it is imperative to develop preclinical assays that can evaluate and aid in mitigating the immunogenicity risk of biotherapeutics. Such evaluations allow for the management of any potential safety, efficacy, and PK risks and to develop safer and more efficacious therapeutics.

**Figure 1:**
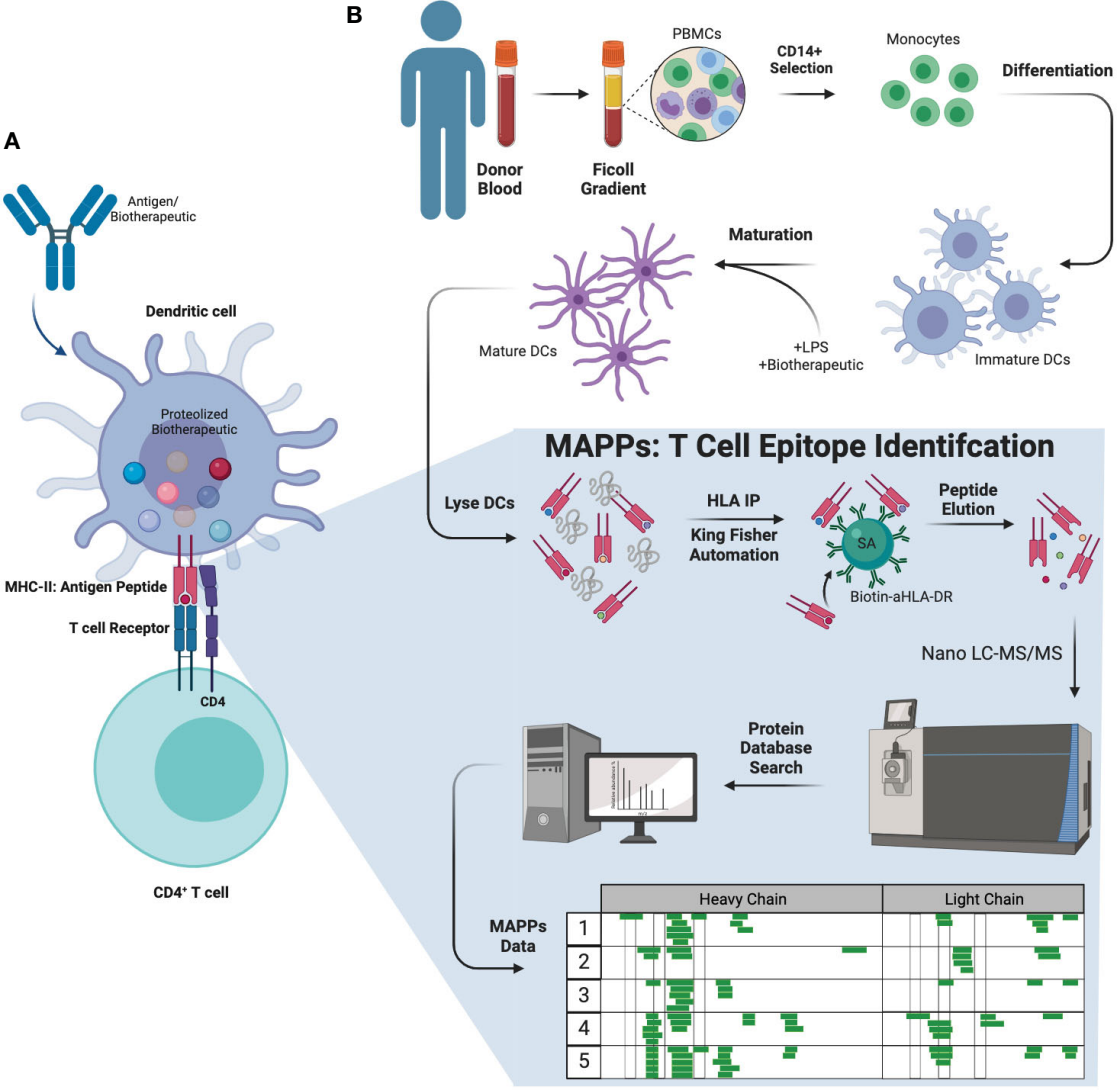
Biology of ADA formation, MAPPs workflow, and therapeutic molecules tested. **(A)** Antigens (or biotherapeutic) were taken up by APCs such as DCs. DCs will process and present the antigen fragments to CD4+ T cells initiating the immune reaction against the antigen, stimulating and leading to the generation of ADAs from B cells. **(B)** MAPPs workflow (ex vivo) used to identify potential T cell epitope(s) that may elicit immunogenicity. PBMCs were extracted from donor blood and selected for CD4+ monocytes. These cells were subsequently differentiated into DCs, which were matured and incubated with LPS and the mAb of interest simultaneously. The cell pellets were lysed and subjected to immunoprecipitation to extract biotherapeutic peptides presented on the DC surface. These samples were injected onto a nano-flow LC-MS/MS using a discovery MS approach, and data was searched against a concatenated forward and reserved human and biotherapeutic database to identify peptide sequences. APC, antigen presenting cell; DC, dendritic cell; LPS, lipopolysaccharide.

Anti-drug antibody (ADA) production is an immune response to biotherapeutic proteins. ADAs are generated *via* one of two mechanisms: T cell dependent or T cell independent B cell activation (5). In the T cell dependent activation of B cell pathway (**Figure 1A**), an antigen (or biotherapeutic) is taken up by antigen presenting cells (APCs) such as dendritic cells (DCs). Upon antigenic uptake through different methods such as phagocytosis, the biotherapeutic undergoes a series of endo-lysosomal proteolytic processing resulting in linear biotherapeutic-derived peptides (6, 7). These peptides are loaded onto a protein complex called major histocompatibility complex (MHC) class II according to the preferential binding profile of an individual’s human leukocyte antigen (HLA) class II alleles. The peptide-MHC complexes are subsequently transported to the cell surface and presented to CD4+ T cells (6, 8), which are then activated and result in downstream B cell activation and generation of ADA (6, 9). ADAs can affect clearance and efficacy of the biotherapeutic via formation of immune complexes and/or neutralization (2) of the biotherapeutic’s ability to engage target molecules. In some cases, ADAs can lead to adverse events, such as infusion related reactions and anaphylaxis (2). The ability to identify potential T cell epitopes that could correlate with increased clinical immunogenicity during early biotherapeutic development could be advantageous in helping select minimally immunogenic candidates.

MHC-associated peptide proteomics (MAPPs, **Figure 1B**) is an *ex vivo* assay used to characterize peptides that are naturally processed and presented on MHC-II molecules by DCs using immunoaffinity enrichment and liquid chromatography tandem mass spectrometry (LC-MS/MS) (4, 10, 11). This approach was initially reported in the early 1990s for the identification of MHC-I associated peptides (12). Increasingly, immunopeptidomics methodologies have been adopted in industry specifically for the assessment of biotherapeutic drug-derived epitopes (4, 10, 11, 13, 14). Over time, developments in sensitive MS instrumentation have enhanced the ability to perform MAPPs (13–17). Our earlier work in semi-automated streptavidin-biotin bead based immunoaffinity (IA) enrichment has allowed us to make further improvements in MAPPs and are presented herein. Previous methods conjugated capture antibodies directly to beads, and immunoaffinity procedures were typically manual, resulting in lower throughput and reproducibility (16). Furthermore, previous methods were typically developed for research purposes and not required to be validated to the extent required for drug development. The field also lacked technical reports and standardized protocols that would allow meaningful comparison of results across different laboratories (18). In summary, there was a need for improved and well-characterized MAPPs methods for implementing in drug development.

Herein, we detail our efforts to systematically evaluate and develop a robust and semi-automated biotin/SA immunoaffinity MAPPs method. We adapted, modified, and optimized the FG nanoparticle beads method from Sekiguchi et al. (13) for higher throughput and robustness by incorporating our semi-automation streptavidin-biotin bead based immunoaffinity capture (19) on a Thermo Scientific KingFisher Flex System. The semi- automated MAPPs method developed was experimentally benchmarked using adalimumab, a drug with high clinical immunogenicity, and compared against other published and publicly available MAPPs datasets. The new MAPPs workflow was also applied to various biotherapeutic molecules with varying clinical ADA incidence rates and showed that biotherapeutics with higher immunogenicity rates consistently presented more peptides and peptide clusters compared to those with lower immunogenicity rates. In these datasets we also detected oxidized and/or deamidated peptides, biotransformations that are known to be associated with elevated immunogenicity (10, 20–22). Most importantly, the method provided highly reproducible and robust MAPPs data across multiple donors, analysts, and days.

## Materials and methods

The MAPPs method is comprised of four main components that were each optimized: (1) dendritic cell generation, maturation, and LPS-induced maturation and antibody pulsing, (2) immunoaffinity (IA) enrichment using streptavidin beads, (3) LC-MS/MS analysis, and (4) data processing. Each part of the method is described below.

### Dendritic cell generation, maturation, and LPS-induced maturation and antibody pulsing

The method used to generate, mature, and stimulate DCs was adapted from Sekiguchi et al. (13) Peripheral blood mononuclear cells (PBMCs) were isolated from fresh peripheral blood of healthy volunteers by density-gradient centrifugation using Uni-Sep blood separation tubes (Accurate Chemical & Scientific Corporation; Westbury, NY). Directly after PBMC isolation, CD14-positive monocytes were isolated using magnetic-activated cell sorting (MACS) anti-CD14 microbeads (Miltenyi Biotec GmbH; Bergisch Gladbach, Germany) according to the manufacturer’s protocol (chrome-extension://efaidnbmnnnibpcajpcglclefindmkaj/ https://www.miltenyibiotec.com/_Resources/Persistent/3c804fa07b66b63215bbacbf43387804b151d77f/SP_CD4.pdf). Monocytes were differentiated into immature DCs in DC medium (RPMI 1640; Thermo Fisher Scientific; Waltham, MA), 10% heat-inactivated fetal bovine serum (FBS; Thermo Fisher Scientific), 1% non-essential amino acids (Thermo Fisher Scientific), 1% Sodium Pyruvate (Thermo Fisher Scientific), 1% Kanamycin (Thermo Fisher Scientific), 3 ng/mL recombinant human interleukin-4 (IL-4; R&D Systems; Minneapolis, MN), 50 ng/mL recombinant human granulocyte-monocyte colony stimulating factor (GM-CSF; R&D Systems) at 3 × 10^5^ cells/mL and maintained at 37°C with 5% carbon dioxide (CO_2_).

After 5 days of culturing, the immature DCs were concentrated in the conditioned culture media by reducing the volume to approximately one-third of the starting volume. The extra media was removed from the plate and centrifuged at 100 × g for 5 minutes to pellet any cells that were within the media. These cells were resuspended in the remaining media and transferred back to the culture plate to minimize cell loss. The immature DCs were matured by adding lipopolysaccharide (LPS) (Sigma-Aldrich; St. Louis, MO) at 1 μg/mL and test antibody at 100 μg/mL for 24 hours at 37°C with 5% CO_2_.

Five different monoclonal antibodies (adalimumab, trastuzumab, bevacizumab, infliximab, and Genetech’s in-house version of bococizumab, mAb A through mAb E, respectively) with varying clinical ADA rates were used for method development and characterization. Adalimumab (mAb A, 5-26% ADA from USPI 2011) and infliximab (mAb D, 2-26% from USPI 2013) were purchased through Caligor Coghlan Pharma. Trastuzumab (mAb B, 0.1% ADA from USPI 2010) and bevacizumab (mAb C, 0.6% ADA from USPI 2009) were internal Genentech research grade commercial molecules. mAb E was an in-house version of bococizumab [48% ADA (23, 24)].

The mature DCs were harvested from the tissue culture dishes by gentle aspiration. The dishes were washed with phosphate-buffered saline (PBS) to remove any remaining cells and combined with the harvested mature DCs. The cells were centrifuged for 5 minutes at room temperature at approximately 300 × g. The cells were then resuspended in cold PBS, counted, divided into aliquots of 2 × 10^6^ cells in 1.5-mL Eppendorf tubes and centrifuged for 5 minutes at 4°C. The supernatant was completely removed, and the cell pellets were stored at -80°C until use.

### Immunoaffinity enrichment with automation - (Sera-Mag streptavidin-coated magnetic beads)

Four different assay formats, two of which utilize direct covalent binding (**Table 1** lines 1 and 2) of the capture antibody versus biotin/SA (**Table 1** lines 3 and 4) were tested for immunoaffinity enrichment to isolate the MHCs and any associated peptides from the cell pellets. Pierce NHS-Activated Magnetic Beads (Pierce Cat.No 88826) and FG NHS BEADS® (Nacalai Cat No.TAS8848N1141) were covalently conjugated with capture antibody via NHS chemistry on primary amines. The FG beads were employed as described by Sekiguchi et al. (13) Pierce NHS-activated magnetic beads were utilized per manufacturer recommendations. Dynabeads® M-280 Streptavidin Beads (Thermo Fisher Cat No.60210), and Sera Mag Magnetic Streptavidin Coated Beads (GE Healthcare Biosciences Cat. No. 30152105010350) bind efficiently with biotinylated capture antibody. Dynabeads and Sera Mag beads were implemented as was done previously (25).

**Table 1:** HLA-DR immunoaffinity enrichment methods comparison.

	Donor	No. of DC (x 10^6^)	Format	Bead Amt (mg)	Ab Clone	Ab Amt (µg)	Target Conjugation Ratio	No. of HC Peptides	No. of LC Peptides	No. of Total Peptides	mAb/Total Peptides (%)	Ab-Bead Chemistry	Comments on Experience
1^^^	A	1	Pierce NHS	3	G46-6	150	NA	ND	ND	ND	ND	NHS	-insufficient sensitivity
2^#^	A	1	FG	1.6	G46-6	50	NA	1	0	15,826	0.0063	NHS	-difficult to acquire beads -beads challenging to use
3	B	2	Dynabeads M-280	3	G46-6	50	20:1	ND	ND	ND	ND	Biotin-Streptavidin	-insufficient sensitivity
4	B	2	Sera Mag	1	G46-6	50	20:1	3	8	25,725	0.042	Biotin-Streptavidin	
5	A	2	Sera Mag	2	G46-6	75	20:1	8	3	12,416	0.089	Biotin-Streptavidin	-easily adapted for automation on the KingFisher -reagents are easier to acquire -reagents arrive in intended condition
6	A	2	Sera Mag	4	G46-6	150	20:1	5	2	12,150	0.058	Biotin-Streptavidin	
7	C	2	Sera Mag	2	G46-6	100	10:1	12	8	29,428	0.068	Biotin-Streptavidin	-G46-6 and GNE generated L243 are comparable
8*	C	2	Sera Mag	2	L243 (GNE)	100	10:1	13	6	28,803	0.066	Biotin-Streptavidin	-automated, consistent biotin incorporation, optimized bead, antibody, & DC amount, consistent peptide & cluster identification
9	C	2	Sera Mag	2	L243 (GNE)	100	20:1	14	5	29,050	0.065	Biotin-Streptavidin	

^ Manufacturer recommendations # Published method (13) * Current optimized conditions PSM, Peptide Spectral Matches. NA, Not Applicable; ND, Not Detected.

The immunoaffinity enrichment of HLA-DR bound peptides was compared using different beads and resin with varying chemistries and sizes. Format number 8 is the optimum automated condition with consistent biotin incorporation, optimized streptavidin bead, capture antibody, and DCs results in consistent biotherapeutic peptide and cluster identification.

In the biotin/SA format, the biotinylated capture antibody was first coupled to streptavidin-coated magnetic beads. Sera-Mag streptavidin-coated magnetic beads (1uM 1% solids, GE Healthcare) were washed twice in 400uL of HEPES-buffered saline containing EDTA and surfactant Polysorbate 20 (HBS-EP; GE Healthcare Biosciences AB) on an automated Kingfisher Flex System (Thermo Fisher Scientific). Details for the KingFisher programming can be found in the **Supplemental Methods**. **Supplemental Methods 1** describes the preparation of the beads. An aliquot of 100 µg anti-human leukocyte antigen-DR (anti-HLA-DR) L243 (26) antibody (Tonbo Biosciences) or G46-6 (BD Biosciences), biotinylated using a target ratio of 10 biotin molecules (5.7 biotin incorporation to L243 as determined by 4’-hydroxyazobenzene-2-carboxylic acid [HABA]) per anti-HLA-DR Ab, was immobilized onto washed Sera-Mag streptavidin-coated magnetic beads by incubating for 2 hours at RT on a thermomixer (Eppendorf) with constant shaking at 2000 rpm. Anti-HLA-DR mAbs were biotinylated by incubating 10 molar equivalents of Sulfo-NHS-LC-biotin (Pierce Thermo Fisher Scientific) to anti-HLA-DR mAb for 60 minutes at room temperature in 10mM sodium phosphate, 150 mM sodium chloride, pH 7.8. Excess unbound biotin was removed using Zeba™ spin desalting column (Pierce Thermo Fisher Scientific) per the manufacturer’s protocol. Biotinylated anti-HLA-DR concentration was determined spectrophotometrically by measuring absorbance at 280 nM using GeneQuant™ 1300 (GE Healthcare).

In parallel, frozen cell pellets were resuspended in 400 μL of lysis buffer (20 mM Tris pH 7.8, 5 mM MgCl_2_, 1% Triton X-100, 1 tablet of cOmplete™ mini EDTA-free protease inhibitor cocktail [Roche Diagnostics GmbH; Mannheim, Germany]), incubated at 4°C for 1 hour with constant shaking, and subsequently centrifuged for 10 minutes at 4°C and 18,407 × g to remove cell debris and insoluble proteins. The cell lysate was then transferred to a 96-well plate and incubated with the streptavidin-biotinylated anti-HLA-DR antibody complex. The samples were incubated at 4°C on a thermomixer with constant shaking at 700 rpm overnight for approximately 16 to 21 hours (13).

Upon completion of the overnight immunoaffinity enrichment, using the **Supplemental Methods 2** KingFisher protocol, the beads were washed twice with 400 μL HBS-EP buffer, twice with 400 μL SN2 buffer (5.0:3.0:0.2:91.8 of 1 M Tris HCl: 5 M sodium chloride: 0.5 M EDTA: Water, v/v/v/v, pH 7.2), and lastly four times with 400 μL water on a KingFisher. Peptides were released through acid elution, incubating the beads-sample complex with 100 μL of elution buffer (2% LC-MS-grade acetonitrile [ACN, JT Baker; Phillipsburg, NJ] and 2% formic acid [Fluka; Germany] in LC-MS grade water [JT Baker; Phillipsburg, NJ]) for 30 minutes at 37°C with gentle shaking at 700 rpm. The supernatant and the magnetic beads were separated. The supernatant was transferred to a Millipore Ultrafree^®^-MC 0.2uM filter unit (Merck Millipore, Ltd.; Ireland), centrifuged for 10 minutes at 21,130 × g and 4°C. The eluent was dried to completeness on a SpeedVac™ (Thermo Fisher Scientific) and subsequently reconstituted in 30 μL of elution buffer immediately prior to LC-MS/MS analysis.

### LC-MS/MS analysis

An Acquity UPLC^®^ M-Class (Waters; Milford, MA) system was used to chromatographically separate and introduce peptides to the mass spectrometer. An aliquot of 15 µL sample, equivalent to 1 x 10^6^ DCs, was loaded onto a C18, 5 μm 100 A, 180 μm × 20 mm trapping column (Waters) with 2% mobile phase B (80% acetonitrile in water with 0.1% formic acid) and 98% mobile phase A (water with 0.1% formic acid) at a constant flow rate of 15 μL/minute for 20 minutes to remove salts and potential interfering components. After trapping, samples were transferred onto the analytical column, an HSS T3, 1.8 μm 100 A, 75 μm × 150 mm (Waters). Peptides were eluted over 75 minutes at 300 nL/min: from initial 5% mobile phase B increase to 20% over 6 minutes, then linearly increased to 40% mobile phase B by 45 minutes and ramped up to 100% mobile phase B at 52 minutes. The gradient was held at 100% B for 8 minutes before returning to initial condition of 5% B over 5 minutes and remained at 5% until 75 minutes to re-equilibrate the column.

The eluant was introduced into an Orbitrap Fusion™ Lumos ™ Tribrid ™ Mass Spectrometer (Thermo Scientific; San Jose, CA) for MS and MS^2^ analysis via a 20 μm EASY-Spray™ nanoflow emitter (Thermo Scientific; San Jose, CA), in positive ionization mode with Ion Spray Voltage of 1.2keV (EASY-Spray™ source, Thermo Scientific; San Jose, CA) and capillary temperature of 300°C. Xcalibur™ 4.1 was used for MS data acquisition; the method consisted of an MS^1^ full scan 60,000 resolution analysis in the orbitrap mass analyzer followed by data-dependent collision-induced dissociation (CID) and higher energy collision induced dissociation (HCD, 15,000 resolution) MS^2^ scan events of the top 15 most intense precursors detected in the full scan with a precursor isolation width of 1.6 mass-to-charge ratio (*m/z*). Data-dependent MS^2^ scans were conducted in the ion trap for CID fragmentation and in the HCD cell (NCE 35) with detection in the orbitrap. An automatic gain control (AGC) target value of 2E5 was used for MS^1^, and 2E4 for MS^2^. Precursors were dynamically excluded for 60 seconds with a repeat count of 5 recurrences in 10 seconds, and only peptides with assigned charge states of two to seven were selected for MS^2^ fragmentation.

### Computational proteomic data analysis

Proteome Discoverer™ 2.2 (Thermo Scientific) with Sequest was used for spectral reduction, database searching and matching mass spectra for biotherapeutic peptide identification from within the human proteome, as well as FDR calculations. Spectra were searched against a concatenated human target-decoy database (www.uniprot.org) with the sequences of test therapeutics (**Supplemental Information**) manually curated. The spectra were searched with no enzyme specificity. Variable modifications of oxidation on methionine residues (+ 15.995 Da) and deamidation (+0.984 Da) on asparagine and glutamine residues were specified, permitting up to four modifications per peptide. A mass tolerance of ± 10 ppm was used for precursors, while a mass tolerance of ±0.5 Da for CID fragmentation with ion trap detection and a mass tolerance of ±0.02 Da for HCD fragmentation with orbitrap detection. Peptide and protein identifications were filtered to a false discovery rate of 1%. XCorr medium and high confidence thresholds used for low- and high-resolution data were the same. XCorr values between 2-2.3, 2.5-3, and 2.8-3.5 were identified as medium confidence for charge states 2, 3, and ≥ 4, respectively. High-confidence peptides have XCorr values greater than 2.3, 3, and 3.5 for charge states 2, 3, and ≥ 4, respectively. The data presented in the study are deposited in the ProteomeXchange MassIVE repository, accession number MSV000093113.

### MAPPs data visualization in heat maps

All of the identified biotherapeutic peptides were plotted onto a heatmap. The heavy chain (HC) and light chain (LC) sequence residues were listed across the top with their respective complementarity determining regions (CDR) annotated in blue. Each green block corresponds to a unique peptide sequence identified, with a color gradient correlating to confidence of peptide identification. Dark green corresponds to high-confidence peptides while light green are medium-confidence peptides. Modifications (biotransformations) are annotated with purple, yellow, and red to indicate methionine oxidation, localized N/Q deamidation, and unlocalized N/Q deamidation, respectively. Some peptides will align in a core region and share a consensus sequence but have ragged N- and C- termini; this group of peptides are a cluster, also known as a nested set.

## Results

### Immunoaffinity enrichment medium is a key component to the sensitivity and robustness of the MAPPs workflow

In this method we focus on the front-end sample preparation steps: cell lysis and specifically implementing biotin/SA bead-based IA enrichment of HLA-DR peptides and its automation on a KingFisher for higher-throughput (HTP) robust sample preparation. Historically, MAPPs methods used agarose (27) or sepharose (14–17, 28–31) beads, and more recently FG nanoparticle beads (13) with capture antibody covalently bounded to the beads, for the immunoprecipitation (IP)/immunoaffinity (IA) enrichment of HLA peptides. Two main aspects of existing MAPPs methodologies are the medium and the crosslinking chemistry. The cell lysis and immunoaffinity enrichment methodology used herein was adapted from Sekiguchi et al. (13) The IA enrichment of HLA-DR bound adalimumab peptides was compared using different beads and resin with varying chemistries and sizes (**Table 1**) (13). As a baseline, the Sekiguchi et al. (13) method was recapitulated as published. The identified peptides and clusters matched previously published data (**Table 1** Ln 1).

Pierce NHS and FG beads form covalent immobilized complexes directly with the capture antibody (a pan anti-HLA-DR) by conjugating primary amines on the N-terminus of protein antibody as well as lysines and arginines. Pierce NHS beads were tested per manufacturer recommendations in which 3 mg of beads were coupled with 150 µg of G46-6. Peptides, which correspond to test therapeutic mAb A were undetectable (**Table 1**, line 1). Using 1.6 mg of FG nano-particle beads coupled to 50 µg of G46-6 (13), 1 and no unique peptides were detected from the HC and LC of adalimumab, respectively (**Table 1**, line 2). On the other hand, streptavidin magnetic beads (M280 Dynabeads and SeraMag) complexed with biotinylated G46-6 provides a streptavidin-biotin spacer between the capture antibody and the bead, theoretically allowing more efficient capture of HLA-complexes by minimizing steric hindrance. This SA/biotin-G46-6 complex immunoaffinity enriched HLA-DR and its bound peptides. No peptides corresponding to adalimumab were detected with M280 Dynabead (**Table 1**, Line 3). SeraMag beads resulted in the detection of 3 and 8 LC mAb peptides (**Table 1**, Line 4). Sera Mag beads resulted in comparable detection of therapeutic peptides as FG nanoparticle beads (**Table 1**, Line 2 vs 4).

### 2 Million DCs, a 10:1 target biotin-to-antibody ratio, 2mg of Sera Mag SA beads, and 100 µg of anti-HLA-DR Ab is optimal for identification of greatest number of presented peptides and clusters

A design of experiment (DOE) style investigation was used to compare and optimize the number of DCs, the amounts of capture antibody, biotinylation incorporation, as well as different capture antibody clones (**Table 1** Lines 5-9) used per sample. The amount of DCs used per enrichment can be varied to modulate the sensitivity of the assay. Increasing the number of DCs to 2 million resulted in more peptide identifications with greater consistency relative to 1 million. However, going beyond 2 million DCs did not result in significant gains of peptide identification (data not shown). Doubling the amount of capture antibody and doubling the amount of beads (maintaining the same bead-to-antibody ratio), in mass, did not improve the assay’s sensitivity as summarized in **Table 1** Ln 5-6.

Biotin challenge ratios were evaluated at 5:1, 10:1, and 20:1 of biotin-to-capture antibody (**Supplemental Figure 1A**). The intact LC-MS spectra indicates a greater number of biotin molecules incorporated when increasing from a 5:1 to a 10:1 ratio. Both 10:1 and 20:1 reagents contained unconjugated antibody, but was more pronounced in the 20:1 reagent. Based on the biotin incorporation, the optimal challenge ratio was between 10:1 and 20:1. In comparing the number of biotherapeutic peptides detected between the 10:1 and 20:1 conjugation ratios, both conditions resulted in comparable number of biotherapeutic and total peptides detected (**Table 1** line 8 and 9). When shown as the percentage of biotherapeutic peptides detected with respect to the total number of peptides, the results are also similar between these two conditions and very low. This is likely a result of the high values observed for the total number of peptides that are not verified through the Percolator algorithm. When the data under each condition is processed with Percolator (**Supplemental Table 1**), the SeraMag condition depicted in line 8 remains the optimal condition.

### Different clones of pan-anti-HLA-DR antibodies were comparable for immunoaffinity enrichment

A commercially obtained pan-aHLA-DR antibody (L243) from Tonbo Biosciences was tested in the MAPPs workflow. L243 had slightly better biotin incorporation than G46-6 (**Supplemental Figure 1B**); the 10:1 and 20:1 conjugation ratios resulted in a greater proportion of mAb more highly conjugated with a median of 5 and 10 for the 10:1 and 20:1 ratios, respectively. L243 and G46-6 perform comparably in the identification of therapeutic specific peptides (**Supplemental Figure 2A**). Tonbo Biosciences’ L243 mAb resulted in the identification of 28 HC and 11 LC peptides across eight common clusters from adalimumab. Genentech produced L243 mAb behaved consistently with the benchmark comparator antibody G46-6. Similar peptides and similar clusters were detected using the different anti-HLA-DR clones (**Table 1** Ln 7-9, **Supplemental Figure 2B**).

### KingFisher automation allows for greater analysis throughput, greater day-to-day and analyst-to-analyst reproducibility, and overall assay robustness

**Figure 2:**
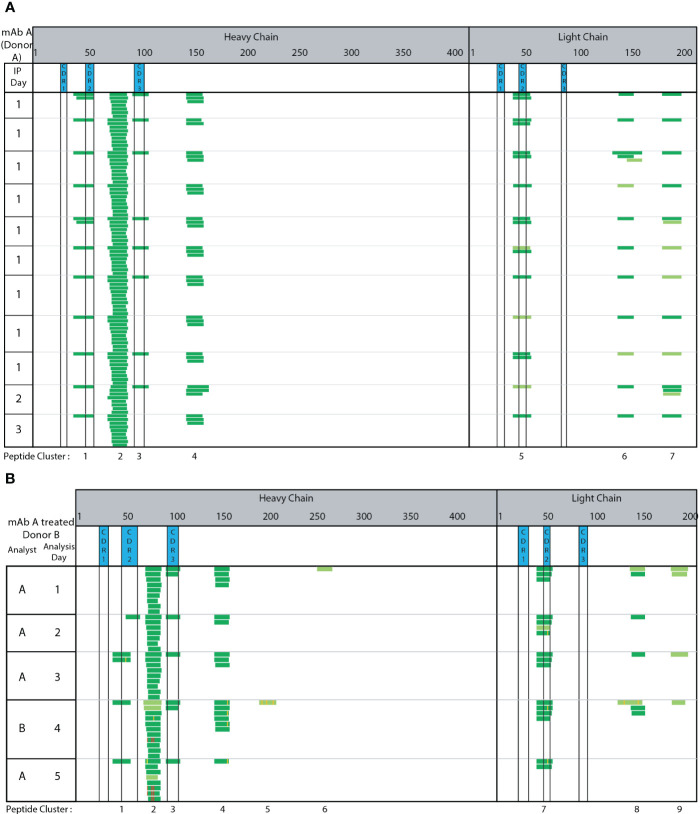
Automated MAPPs is a robust assay. **(A)** Day-to-day variability **(B)** Analyst-to-analyst variability. Dark green corresponds to high confidence identification. Light green corresponds to medium confidence identification. Modifications are annotated with purple, yellow, and red to indicate methionine oxidation, localized N/Q deamidation, and unlocalized N/Q deamidation, respectively.

The complexing of the capture Ab to SA beads, washing, and IA enrichment steps were automated using a KingFisher Flex System. We automated these steps by extrapolating from our extensive experience in developing highly-sensitive quantitative bioanalytical methods for ADCs (19, 32), which utilize the biotin/SA magnetic bead platform for immunoaffinity capture out of various biological matrices. Seven peptide clusters were identified consistently across 11 technical replicate runs of adalimumab treated monocyte derived DCs generated from the same donor PBMC from a single blood draw and analyzed across three different days (**Figure 2A**). The peptides and clusters identified include adalimumab specific peptides spanning HC CDR2, HC CDR3, and LC CDR2 regions (clusters 1, 3, and 5, respectively). Two clusters (cluster 2 and 4) were detected from the HC framework and constant regions and 2 clusters (cluster 6 and 7) were detected from the LC constant regions. Greater than 95% consistency was observed in peptide and cluster identification across these 11 replicate samples prepared and analyzed across three different days. Using adalimumab, the automated MAPPs method is reproducible and robust through intra-day analysis (**Figure 2**, IP day 1) and inter-day analysis (**Figure 2**, IP days 2 and 3).

The assay’s robustness and consistent performance between analysts was assessed by comparing peptides and clusters identified from five replicate adalimumab treated DC pellets generated from a single donor. Two different analysts (A & B) prepared and analyzed the samples across five days (**Figure 2B**). Four clusters (2, 3, 4, and 7) were consistently detected between all analyses. Two clusters (3 and 7) were in the CDR regions (HC CDR 3 and LC CDR2). Cluster 1 and 8 were observed in 80% (4 of 5) of samples. These results demonstrate automated MAPPs significantly minimizes any impact/influence from analyst-to-analyst variability. Minimal day-to-day variability is observed over the five-day period. Therefore, in using this semi-automated MAPPs method with different donors, any differences observed in peptide and cluster presentation can be confidently attributed to donor-to-donor differences, including HLA allele polymorphisms, and/or biotherapeutic differences as opposed to method variability.

### Using semi-automated MAPPs, 5-10 donors are sufficient to observe indicative peptides and clusters

**Figure 3:**
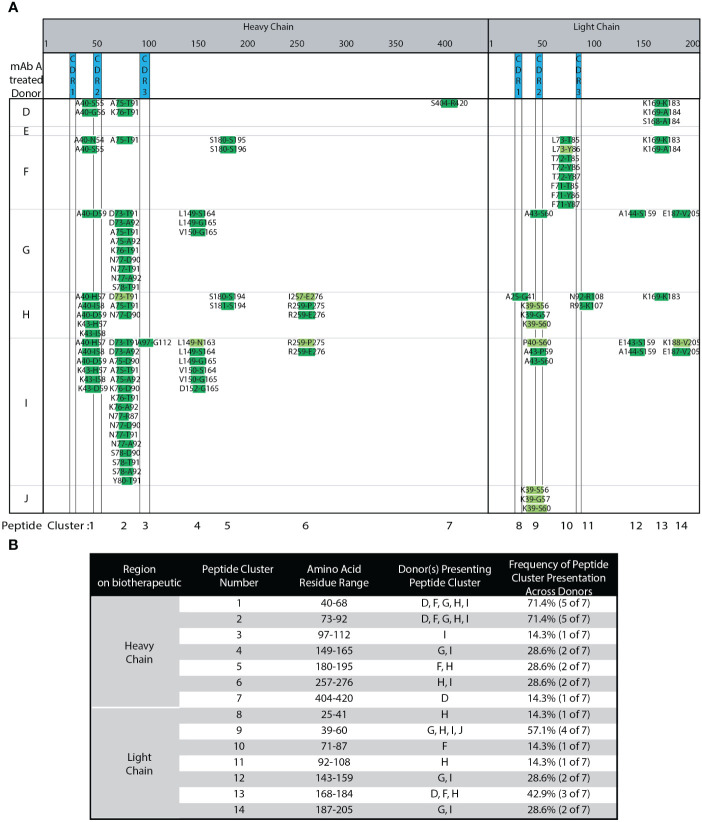
Adalimumab Donor-to-donor variability. **(A)** Heat Map of adalimumab (mAb A) peptides presented across 7 donors. **(B)** Summary of adalimumab HC and LC peptides, peptide clusters, and their presentation frequencies amongst the 7 tested donors detected in **(A)**.

DCs from seven different, randomly selected donors were treated with adalimumab. The heat map in **Figure 3A** summarizes the different peptides and clusters observed across donors. Compared to our robustness analysis (**Figure 2**), many of the same peptides and clusters were observed in the seven new donors. **Figure 3B** summarizes the different clusters identified along with the donor DCs that present peptides in each cluster, and the frequency of peptide cluster presentation across the donors. Six out of seven donors presented published peptides and clusters (13, 15). Approximately 30% biological variability was observed in 2 out of 7 donors, where less than two peptide clusters were presented. One donor (J) presented only 3 peptides that were only from LC CDR2 and with only medium confidence. Peptides unique to adalimumab were detected in over 50% of donors. These unique peptides map to HC CDR2 (cluster 1) and LC CDR2 (cluster 9). Cluster 2, has a shared peptide core ranging from HC residue 73 to 92, was detected in 5 of 7 donors (71.4% of donors). The DCs in this experiment were from donors that contain HLA alleles representative of the population (33). Combining our robust, semi-automated MAPPs method with population representative DCs, we find a minimum of 5 donors, ideally 10, are needed per biotherapeutic analyzed.

### MHC-II presented peptides detected from the biotherapeutic and self proteins have a distribution of peptide lengths consistent with canonical class II presented peptides

The distribution in the length of identified biotherapeutic-related peptides along with those from endogenous proteins were plotted in **Supplemental Figure 3**. Using the donor-to-donor variability dataset, a histogram analysis revealed that 16 amino acids was the median length of identified biotherapeutic-related peptides (**Supplemental Figure 3A**) and non-therapeutic self-peptides (**Supplemental Figure 3B**). This range of peptide length is consistent with reported median peptide length and distribution of peptide lengths for MHC class II peptides (8, 27, 28, 34, 35).

With our automated MAPPs method, we implemented washes to minimize non-specific binding and also regularly assessed the number of peptide and protein IDs other than the biotherapeutic (data not shown). While we were not able to completely eliminate non-specific binding, we were able to identify and confirm the non-biotherapeutic peptides. The identity of these peptides come from proteins such as the various isoforms of cathepsin along with CLIP peptide (portion of the invariant chain). The various forms of cathepsin are responsible for proteolyzing the biotherapeutic. The CLIP peptide resides in the peptide binding groove until it is displaced by a potential immunogenic epitope for presentation to the T Cell receptor. These additional non-biotherapeutic peptide-to-protein identifications are as expected based on the canonical understanding of the presentation pathway. Steiner et al. have demonstrated the utility of using these non-biotherapeutic peptides to normalize data sets and how it can be used to assess the quality of MAPPs data (14).

### Biotherapeutic peptide and cluster presentation trends with clinical ADA rate

**Figure 4:**
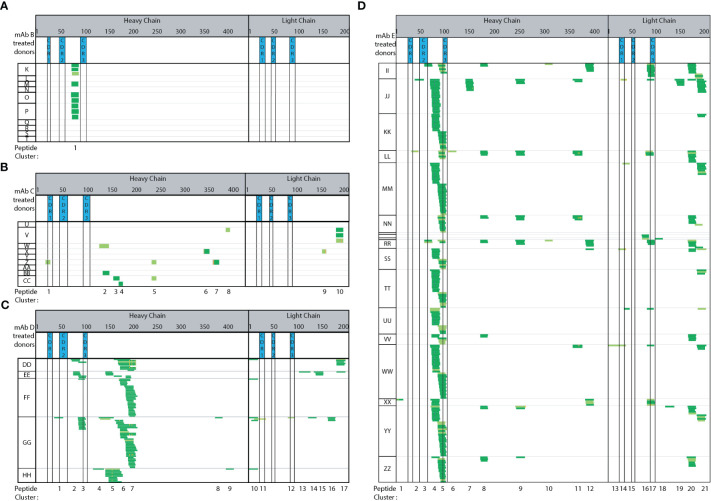
Immunogenic mAbs contain higher number of potential CD4 T cell epitopes. Heat maps summarizing automated MAPPs analysis of **(A)** trastuzumab (mAb B), **(B)** bevacizumab (mAb C), **(C)** infliximab (mAb D), and **(D)** Genentech’s in-house generated bococizumab (mAb E), in the order of low to high ADA rates. The reported clinical ADA rates are summarized in Material and Methods.

Four additional mAbs (trastuzumab, bevacizumab, infliximab, and Genentech’s version of bococizumab, mAbs B-E, **Figure 4**) were tested using semi-automated MAPPs. Each mAb has varying modes of action (MOAs) and clinical ADA rates. Each mAb was tested using a minimum of five healthy donors from which DCs were obtained. These mAbs have a range of reported clinical immunogenicity incidence rates, ranging from 0.1% (low ADA) to 48% (high ADA), summarized in Materials and Methods.

Adalimumab has a reported clinical ADA rate of 5-26% (USPI 2011) while trastuzumab (**Figure 4A**) has a clinical ADA rate of 0.1% (USPI 2010). Adalimumab treated DCs (**Figure 3A**) presented 14 peptide clusters overall across 7 donors, while trastuzumab treated DCs presented one peptide cluster (**Figure 4A**). The singly identified trastuzumab cluster is the same HC framework peptide cluster 2 in adalimumab. The amino acid sequence ranging from residue D73 to K98 (or R98 for trastuzumab) differ by 4 amino acid residues, an 84% sequence homology (**Supplemental Sequence Alignment A**). Even though peptides unique to the therapeutic were detected from adalimumab, there were also many peptides and clusters that are from the constant and framework regions. These regions are not unique in sequence (adalimumab vs trastuzumab).

Bevacizumab (**Figure 4B**) also has a relatively low ADA incidence rate of 0.6% (USPI 2009). The number of peptides detected are minimal and sporadic across donors. There are more peptides and clusters detected when DCs were treated with bevacizumab (15 peptides, 10 clusters), as compared to trastuzumab (9 peptides 1 clusters) (**Figure 4A** vs **4B**). In comparing bevacizumab to adalimumab, there are fewer and more disparate peptides and clusters from bevacizumab (**Figure 4B** vs **Figure 3A**, respectively). Infliximab has a moderately high ADA incidence rate of 2-26% (USPI 2013). A total of 17 clusters were detected across five donors (**Figure 4C**). Two clusters (1 and 3) are from the HC CDR2 and CDR3 regions and two clusters (11 and 12) are from the LC CDR1 and CDR3 regions. The majority of the peptides detected are from the HC and LC constant regions. In comparison to adalimumab, the HC constant region (starting at adalimumab D109 to terminal K451) shares 90% sequence homology with mAb E and 99% sequence homology with trastuzumab, bevacizumab, and infliximab (**Supplemental Sequence Alignment B**). When compared to adalimumab, the LC constant regions (starting at mAb A T97 to terminal C214) shares 100% sequence homology to trastuzumab and bevacizumab, 97% sequence homology to infliximab, and 99% sequence homology to mAb E (**Supplemental Sequence Alignment C**).

Of the five mAb molecules tested, mAb E has the highest ADA incidence rate of 48% (23, 24). Across 18 donors, 21 peptide clusters were detected. Clusters 4, 5, and 17 are the most predominantly presented from the HC framework, HC CDR3, and LC CDR3 regions, respectively. The compilation of mAb E heatmaps is a thorough assessment of donor-to-donor variability, day-to-day variability, as well as analyst-to-analyst variability (**Figure 4D**). The mAb E summary heat map is generated by two analysts, across 4 days, using DCs from 18 donors. Additionally, methionine oxidation along with N and Q deamidation were detected. The observed biotransformations were consistent across donors, sample preparation days, and multiple analysts.

Overall, a greater number of peptide clusters (**Supplemental Figure 4**) were observed for molecules in which there is a higher reported clinical ADA rate. Additionally, more peptides and clusters were detected from the biotherapeutic-specific regions, such as HC and LC CDRs, of mAbs with higher ADA. No peptides or clusters were detected in CDR regions from trastuzumab. A single bevacizumab HC CDR1 peptide was detected from a single donor. Conversely, adalimumab, infliximab, and mAb E that have higher ADA rates, all presented CDR peptides. Adalimumab presented peptides from HC CDR2 and CDR3 (71% of donors and 14% of donors) and LC CDR1, CDR2, CDR3 (14%, 57%, and 14% of donors, respectively). Infliximab presented peptides from HC CDR2, CDR3 (20% and 60% of donors, respectively) and LC CDR1, CDR3 (20% of donors for both). Lastly, mAb E with the highest ADA rate presented HC CDR2 (17% of donors), HC CDR3 (72% of donors) along with LC CDR1 (22% of donors), LC CDR3 (39% donors) peptides consistently. Overall, we observed peptide and cluster presentation (**Supplemental Figure 4**) as well as therapeutic specific CDR regions, trends with clinical ADA incidence rates.

### Automated MAPPs allows for confidence in the detection and identification of biotransformations

Methionine oxidation (Met-Ox), Asparagine (N) and/or Glutamine (Q) deamidation are modifications observed on a portion of the presented biotherapeutic peptides detected by MAPPs. Four observations were made in relation to the detected biotransformations. First, the paired native unmodified peptides were detected along with the modified version of the peptide. Second, the biotransformed residue(s) were detected in multiple peptides that share the same core, but having different N and C-termini resulting in varying peptide lengths. For example, N/Q deamidation is observed in multiple unique peptides within cluster 2 (**Figure 2B** analyst A day 5). N/Q deamidation in cluster 4 is another example. Third, the same modified residue was observed in subsequent DC sample preparations that were generated from the same donor. In **Figure 2** analyst B-day 4 and analyst A-day 5 both detect N/Q deamidation in the HC clusters 2, 4, and 7. Two analysts on two different days were able to identify the same peptides in both the native and modified forms. Lastly, the same modification was detected across multiple donors. In the case of infliximab, the deamidation on the same residue was observed in 2 out of 5 donors (DD and GG cluster 7, **Figure 4C**). Met-Ox on mAb E was presented in 44% of donors (8 of 18: JJ, KK, MM, SS, TT, UU, WW, YY in cluster 4). These four observations further demonstrate the consistency, sensitivity, and robustness of our automated MAPPs assay.

## Discussion

### Capture format (immobilization chemistry, capacity, Ab clones) is critical to MAPPs assay sensitivity

Immunoprecipitation inefficiencies with sepharose beads led Sekiguchi et al. to develop a MAPPs method that utilizes FG nano-particles (13). However, we found some limitations with this approach. The Sekiguchi method is a multi-day process in which beads do not disperse easily, required manual preparation of samples, and a significant amount of finesse was required for consistent performance. First, multiple times the FG nanoparticles dried out while in transit from vendor to bench, resulting in compromised antibody coupling. Second, IP efficiency was impacted by the FG nanoparticle’s tendency to clump. If FG nanoparticles clumped, then extremely low numbers of peptides to no peptides were detected. Thus, delicate handling, balancing sonication, manual inversions and gentle vortexing, along with utilizing a precise ice-to-water bath ratio was necessary to ensure consistent performance. Thirdly, longer sample preparation time could result in artificial sample preparation-induced modifications, thus confounding the interpretation of identified biotransformations. Lastly, due to the sensitive handling conditions of the FG nanoparticles, this format was not conducive for transfer onto a high-throughput automated system, such as a KingFisher. Due to these challenges, we endeavored to develop an assay which would address each of these concerns while retaining sensitivity and introduce robustness and reproducibility. The first consideration was bead and resin comparison. The Sekiguchi et al. (13) method used FG beads. Due to the challenges described above, we explored other bead options that were more readily available commercially and suitable for automation implementation (**Table 1**).

We considered a coupling reaction successful when >75% of the antibody is conjugated to the solid phase based on manufacturer specified binding capacity. Whether using FG nanoparticles or Pierce NHS beads, we found coupling the capture antibody directly to the capture medium via NHS chemistry to consistently be less than this, based on measuring total protein content *via* nano drop A280 measurement prior to and post coupling. Directly coupling the capture antibody to the beads non-specifically conjugates it to primary amines (N-terminus, lysine side chain, and arginine). This non-specific conjugation to the bead may not be conducive to optimal MHC capture. The binding site on the capture antibody could become completely obstructed, sterically hindered or the orientation of the Ab on the bead may be in a direction that hinders MHC capture.

As an alternative, we considered a streptavidin-biotin based immunoaffinity enrichment approach, routinely utilized for automated immunoaffinity capture of antibody drug conjugate (ADC) bioanalysis (19, 32). This approach provides a streptavidin-biotin spacer between the capture antibody and the bead, theoretically allowing more efficient capture of HLA-complexes by minimizing steric hindrance. Biotin is also less likely to disturb the natural function of the molecule it is conjugated to due to its small size. Biotin and streptavidin bind with extremely high affinity, with a fast on rate and high specificity. Dynabead M280 and Sera-Mag beads were used in this work to test streptavidin-to-biotin coupling chemistry.

Capacity of the capture medium is also a vital point of consideration in selecting the capture format. Dynabead M280 (2.8 µm) and Sera-Mag (1 µm) beads employ the same methodology and chemistry, differing only in the bead size. The difference in size translates to a 4-fold capacity difference between M280 and Sera-Mag beads. By using a smaller bead size, the surface area-to-volume ratio results in greater capacity, therefore, increased sensitivity (25). We observed more therapeutic peptides using Sera-Mag beads as compared to M280 (**Table 1**, Ln 3-4). We believe that the greater bead capacity allowed for greater sensitivity that resulted in the detection of more therapeutic peptides. Hence, Sera-Mag beads are preferred for their greater sensitivity. Per manufacturer specifications, FG nano particles are 2x smaller than Sera-Mag beads (200 nm vs 1um, respectively). FG beads have a binding capacity of approximately 200,000-300,000 pmol/mg, whereas Sera-Mag beads have approximately 5600 pmol/mg binding capacity. Theoretically, FG beads have 40-fold greater binding capacity than Sera-Mag beads. However, we have shown that even with lower theoretical binding capacity, Sera-Mag beads are able to perform comparably while fulfilling the criteria of ease of use and adaptability to HTP sample preparation. When taking into consideration coupling efficiency, capture capacity, overall ease of use and availability, the ability to automate for HTP analysis, as well as overall cost, Sera-Mag was the optimal capture medium of the different tested formats. Furthermore, Sera-Mag bead enrichments performed reproducibly and robustly in conjunction with the KingFisher automation.

For simplicity, robustness, and consistency, the lysis buffer was modified to contain the commercial Roche inhibitor cocktail rather than making fresh specific protease inhibitors with every analysis, as described by Sekiguchi et al. (13) Contrary to Sekiguchi et al.’s experience (13), an increase in background noise or interference in the mass spectra with this change was not observed (data not shown). Utilizing an automation platform enables robust and consistent wash steps. We hypothesize our automated wash steps allow us to utilize cocktail inhibitor tablets without additional background interference.

### HTP automation enabled greater assay robustness and larger screening panels

HTP automation affords the incorporation of robust, thorough washing at various steps in the workflow. We optimized the washes on the KingFisher to minimize non-specific binding and demonstrated that the semi-automated MAPPs method could achieve the same level of sensitivity as the higher capacity FG nanoparticle beads, based on a comparable number of HC and LC peptides. Automation of the wash steps allowed more washes with more stringent buffers than the Sekiguchi method, enabling the use of protease inhibitor tablets, which ultimately simplified the lysis buffer. The automated washes resulted in an overall cleaner MS background.

We demonstrate automated MAPPs to be robust through inter- and intra-day analyses (**Figure 2A**), as well as through consistency between analysts (**Figure 2B**). Our automated MAPPs method was benchmarked against other published methods (4, 13, 15). We observe the same HC CDR1 and LC CDR2 clusters along with other HC and LC framework and constant regions. Upon comparison with Sekiguchi et al.’s method, we observe the same cluster 2 (HC framework 3 region between CDR2 and CDR3) presenting more peptides. Similarly, with HC CDR2, LC CDR2 and LC constant regions.

Previous MAPPs assays used to characterize biotherapeutic-derived peptides that are naturally processed and presented on MHC-II receptors by DCs use a range in the number of DCs, 1 to 5.4 x 10^6^ DCs (4, 13, 14, 36). Our semi-automated MAPPs method has been optimized to perform robustly and reproducibly with 2 x 10^6^ cells. Due to the ability to process multiple samples in parallel, automation enabled larger screening panels. This translates to the ability to compare multiple test molecules with multiple donors in a single panel and preparation, increasing statistical power. By including multiple molecules with the same target pathway, one is able to better understand whether immunogenicity is molecule sequence specific or influenced by the target and its expression on APCs. For example, a panel of aPD1/aPD-L1, such as nivolumab, pembrolizumab, atezolizumab, durvalumab, and avelumab, can be cross compared. If the entire panel showed a greater number of presented peptides and clusters, then it could be an effect of augmented internalization and presentation versus being attributed to the molecule’s primary sequence. Additionally, automated MAPPs can be used in lead candidate molecule selection or to engineer out higher-risk target sequence(s) (4, 13, 37). HLA genetic diversity is vast as they are highly polymorphic genes (38, 39), therefore, the ability to potentially sample more donors within a panel also increases the likelihood that MAPPs results for immunogenicity risk assessment may be representative of the larger population and the HLA genotype variability and diversity.

The robustness of our automated MAPPs method has allowed us to further characterize donor-to-donor variability. Compared to previously published adalimumab MAPPs data from Karle et al. (15), Sekiguchi et al. (13), and Meunier et al. (40) (n=2-10), 6 of 7 donors tested presented published peptides and clusters. Karle states that approximately 20 donors are required to cover most of the presentable sequence regions (10) due to differential peptide presentation by different donors based on their HLA class II alleles. MAPPs has historically been a resource-intensive, laborious multi-day workflow that requires a large number of DCs, biotherapeutic, and reagents; thus, MAPPs is not conducive to routine large donor panel analyses. Using the semi-automated MAPPs method, approximately 30% natural biological variability (2 of 7 donors) was observed, where less than 2 peptide clusters were observed. With our streptavidin bead-based semi-automated MAPPs method, we have demonstrated improved robustness and reproducibility, and minimized the need for experiments with a large number of donors. If donor HLA alleles are representative of the majority of the population (33), we find a minimum of 5 donors, but ideally 10, are necessary for a panel to be indicative of identifying potential immunogenic epitopes (**Figures 3**, **4**).

### Alternate HLA-DR capture Abs can be implemented with automated MAPPs, with possible extension to DP, DQ, and pan-HLA capture Abs

The complexity of the MHC immunopeptidome in the human population is amplified by the highly polymorphic and structurally different HLA molecules-DR, DP, DQ. The allelic diversity can alter the structure and specificity of the peptide-binding sites of the HLA molecules (18). HLA class II molecules are α/β heterodimers encoded by the highly polymorphic genes, HLA-DR, HLA-DP, and HLA-DQ. The human genome comprises over 10,000 different HLA allelic forms and each person expresses typically 8 different class II allotypes, resulting in a vast HLA peptidomic complexity at the population level (18, 41). It is understood that T cell responses against allergens are associated with HLA-DR and are rarely associated with HLA-DP and HLA-DQ (42, 43). Our automated MAPPs method, along with most published reports, use a pan anti-HLA-DR mAb. The HLA-DR alpha chain is more conserved across different genotypes as compared to DP and DQ (38), hence why different pan-DR capture antibodies work well across the board. The expression level of HLA-DP and DQ differ from HLA-DR (44) and both are believed to play a minor role in the context of biotherapeutic immunogenicity; however, the exact reason is not fully understood in the context of *in silico* prediction (45). Additionally, there are few examples in current literature demonstrating an association between HLA-DP, DQ, and immunogenicity of biotherapeutics (46). It should be noted that the collective dataset presented herein are from donors that cover 21 of the 26 HLA class II (DR, DP, and DQ) alleles that are most frequent in the general world-wide population (33). Further investigation into HLA-DP and HLA-DQ peptide presentation may provide insights into furthering our understanding of biotherapeutic immunogenicity.

Using the same optimized immunoaffinity capture conditions, we were able to substitute in different pan anti-HLA-DR antibody clones and successfully detect and identify presented peptides. This result suggests the potential ease of further applying the biotin/SA methodology to additional capture antibodies and opens up the possibility of gaining a more integrated view of potential immunogenic biotherapeutic epitope presentation by including HLA-DP and DQ. However, it is important to note that with each antibody substitution, there may be a need to reoptimize assay parameters, such as biotin conjugation ratio, amount of antibody and wash conditions.

### Robustness of automated MAPPs enables consistent detection of biotransformations

Intrinsic factors such as structural homology with respect to human amino acid sequence (47) and biotransformations (10, 20), can contribute to the unwanted immunogenic potential of therapeutics (9, 21, 46, 48, 49) and can have direct or indirect effects on immunogenicity. The modified part of the biotherapeutic itself could induce an immune response, or its presence can affect the tertiary structure of the protein subtly causing the biotherapeutic to become immunogenic (21).

In our datasets Met-ox as well as N and/or Q deamidation (**Figures 2** **–** **4**) were detected along with the corresponding native peptides. Deamidation contributes to charge heterogeneity and together with oxidation both have been associated with potential risk of enhanced immunogenicity (21). Deamidation can be accompanied by some degree of oxidation, conformational changes, fragmentation and/or aggregation, posing a risk of enhanced immunogenicity (21). Oxidative chemical modification of amino acid residues alters secondary and tertiary protein structures (22). This favors interaction between protein surfaces and subsequently leads to noncovalent aggregation (22). Shorter sample preparation time, from cell lysis to IA capture, elution, and LC-MS/MS analysis, decreases the probability of sample handling induced biotransformations, although its potential cannot be entirely eliminated. Using this robust automated MAPPs workflow enabled the confident detection, identification, and localization of biotransformations consistently across various biotherapeutics tested across multiple donors, days, and analysts.

### Biotherapeutic peptides and cluster presentation may be indicative of clinical immunogenicity incidence

Framework region 3 is a common cluster observed in most tested mAbs irrespective of their clinical ADA rates. The prevalent display of peptides in this region has also been observed by other labs as well (4, 13, 15), irrespective of the test molecule’s reported ADA rate. Aside from the common presentation of promiscuous regions, we also find that a greater number of peptide clusters were observed for molecules with higher reported clinical ADA rates. Walsh et al. also observe that immunogenic mAbs contain higher number of potential CD4 T cell epitopes (4). In our datasets, more peptides and clusters were detected from the molecule-specific regions, such as HC and LC CDRs, of mAbs with higher ADA rates. Similar to Walsh et al’s (4) observation, a marked difference in the number of clusters overlapping CDR regions that displayed the highest sequence diversity were observed between non-immunogenic control mAb and immunogenic mAbs. However, additional factors could also contribute to a deeper understanding of MAPPs data such as a molecule’s mode of action that influences APC internalization and subsequent presentation. Furthermore, the consistency of cluster presentation across donors in a panel is important in determining whether a biotherapeutic may be classified as having low versus high risk of producing an immune response. Trastuzumab consistently only presented cluster 1 across four donors. Adalimumab, infliximab, and mAb E present larger number clusters- 7 to14, 17, and 21, respectively. Donors present bevacizumab (0.6% ADA) peptides sporadically, resulting in a greater number of clusters (10), however, the clusters are not consistently presented across donors. The frequency of cluster presentation is a trait of MAPPs that could impact interpretation of data. Another variable to consider is how a peptide with nearly 100% sequence homology can be presented from two different molecules with vastly different clinical ADA rates. This observed phenomenon potentially suggests that there may be a quantitative component that could be complementary to the interpretation of MAPPs data. If MAPPs results are interpreted only in a qualitative manner to identify potential immunogenic sequences, there is likely unrealized additional value in a semi-quantitative means of meta-data analysis to determine the extent of peptide presentation and if clinical ADA is better correlated to achieving a particular threshold of peptide presentation rather than just presence or absence of presentation. The general observation of clinical ADA rate trending with peptide and cluster presentation suggests that there may be potential to use overall presentation (eg, number of clusters, number of peptide spectral matches, number peptides-unique or normalized per donor, or the amount of biotherapeutic specific peptide presentation as a percentage of the total number of peptides presented) as an immunogenicity risk assessment tool.

## Conclusion

MAPPs is a qualitative method that combines immunoaffinity enrichment with LC-MS/MS peptidomics. Previous versions of MAPPs assays were limited in robustness, ease of use, and limited in the ability to automate, often leading to varying results between laboratories (18). To overcome these challenges, we have developed a robust, sensitive, novel, and reproducible semi-automated MAPPs assay using a streptavidin bead-based immunoaffinity enrichment approach. The assay enriches HLA-DR peptide complexes out of cell lysate *via* automated immunoaffinity capture on a KingFisher Flex System and subsequently releases bound peptides through acid dissociation. The released peptide milieu was analyzed and identified via nano LC-MS/MS. The inter- and intra-day consistency of this automated MAPPs assay, as well as the analyst-to-analyst comparison, demonstrate the robustness of the method. The robustness of this assay allowed for confident assessment of donor-to-donor variability; a minimum of 5 donors (ideally 10) per panel is necessary to be representative of the population. The robustness of this automated assay enabled the confident identification of biotransformations (oxidation and deamidation) that may be pertinent to eliciting an immunogenic response; however, their significance remains to be further investigated. This assay was tested with a panel of 5 benchmarking biotherapeutics with a range of reported clinical ADA incidence rates (0.1 to 48%). A high frequency of peptide and cluster presentation is observed for moderate to high immunogenic molecules while minimal presentation is observed for low immunogenic molecules. These analyses used less than half the number of cells as compared to pertinent published literature. This assay has been one of our immunogenicity risk assessment tools to support health authority communications of molecules in early development and beyond. The adaption and implementation of MAPPs to assess the potential immunogenicity of biotherapeutic drug-derived epitopes has increased (11); thus, it is important to assess additional biotherapeutics (ie, mAbs, proteins, unique formats) for better understanding of MAPPs’ true predictive ability. The automation of MAPPs analysis has enabled higher throughput, thus the ability to incorporate this as one of the standard immunogenicity screening assays. The robustness and reproducibility gained through automation coupled with higher throughput enables confident comparisons across multiple therapeutic candidates as well as sampling a more representative patient population.

## Data availability statement

The data presented in the study are deposited in the ProteomeXchange MassIVE repository, accession number MSV000093113.

## Ethics statement

The human samples used in this study were acquired from Genentech Samples for Science program. Blood was obtained from consenting anonymous donors through Genentech's Samples for Science program for research conduct. The studies were conducted in accordance with the local legislation and institutional requirements.

## Author contributions

ML: Conceptualization, Formal Analysis, Supervision, Writing – original draft, Writing – review and editing. OS: Conceptualization, Supervision, Writing – original draft, Writing – review and editing. SW: Data curation, Formal Analysis, Writing – original draft, Writing – review and editing. JL: Data curation, Writing – review and editing. LK: Data curation, Writing – review and editing. BO: Data curation, Writing – review and editing. RM: Data curation, Writing – review and editing. AH: Data curation, Writing – review and editing. SC: Writing – review and editing. SK: Writing – review and editing, Conceptualization.
