# Supplementary material for: Development of a semi-automated MHC-associated peptide proteomics (MAPPs) method using streptavidin bead-based immunoaffinity capture and nano LC-MS/MS to support immunogenicity risk assessment in drug development
Source: Front Immunol. 2023 Nov 10;14:1295285. doi: 10.3389/fimmu.2023.1295285 (PMC10667718; doi:10.3389/fimmu.2023.1295285)

**-Supplemental Information:**

**Biotherapeutic Sequence Alignment** of (**A**) Heavy Chain constant region 1 (CH1) shows 84% sequence homology between adalimumab vs trastuzumab, 80% compared to bevacizumab, while only 60 and 52% homology with infliximab and Genentech’s in-house generated bococizumab (mAb E), respectively.

**A.**

**
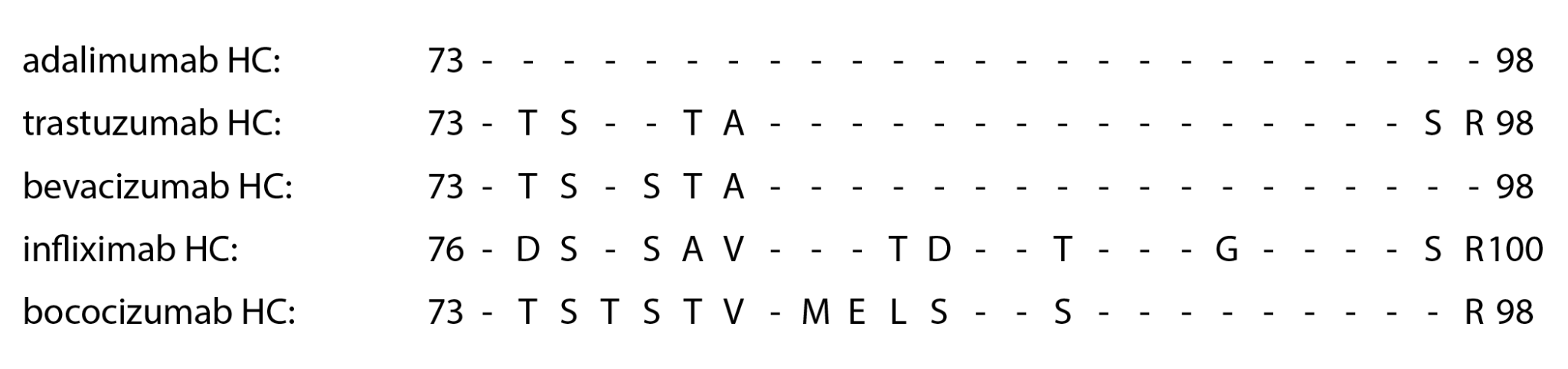
**

(**B**) Heavy Chain constant regions 2 and 3 (CH2 and CH3) in the Fc are 99% homologous between adalimumab, trastuzumab, bevacizumab, and infliximab, but only 90% homologous with Genentech’s in-house generated bococizumab (mAb E).

**
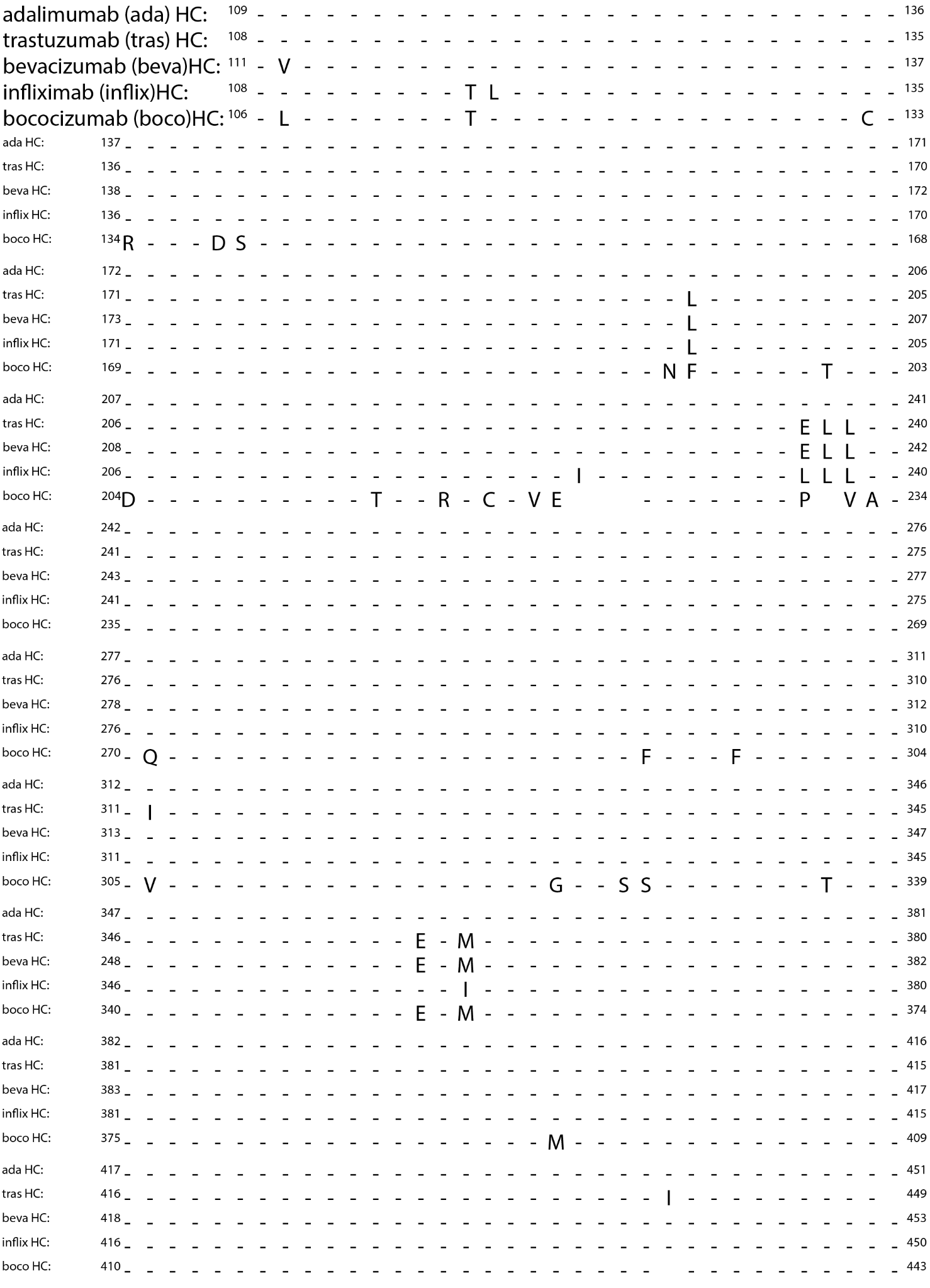
**

(**C**) Light chain constant region (CL) is 100% homologous between adalimumab, trastuzumab, and bevacizumab, but only 97% and 99% homologous with infliximab and Genentech’s in-house generated bococizumab (mAb E), respectively.

**
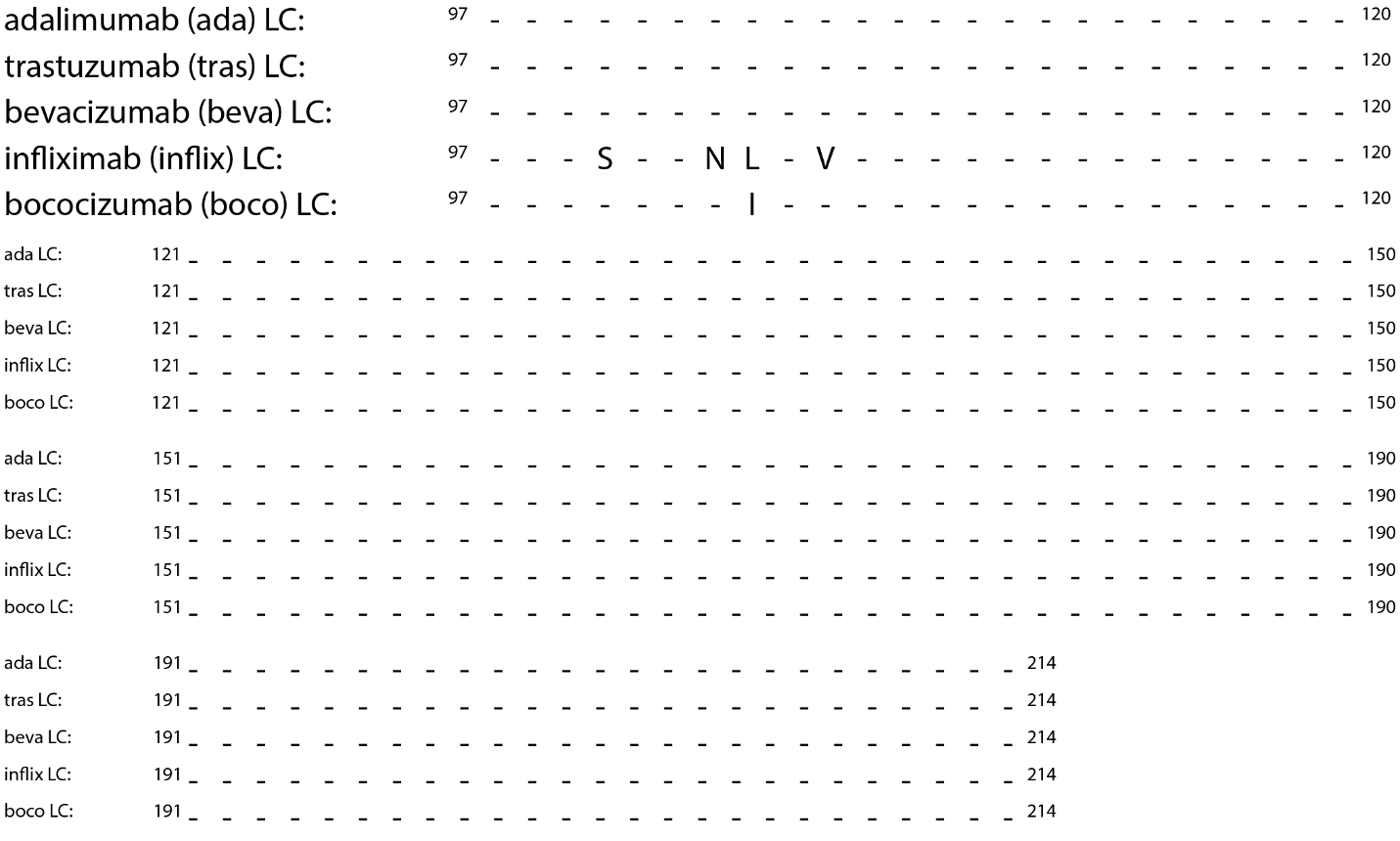
**

**Supplemental Table 1.** HLA-DR immunoaffinity enrichment comparison incorporating Percolator for machine-learning based peptide verification, in place of fixed value PSM. Nine HLA-DR immunoaffinity enrichment formats were analyzed. The number of HC, LC, and total peptides are summarized along with the percentage of biotherapeutic peptides with respect to the total number of peptides. Format number 8 is the optimum condition and has the highest percentage of biotherapeutic-to-total peptides.

**
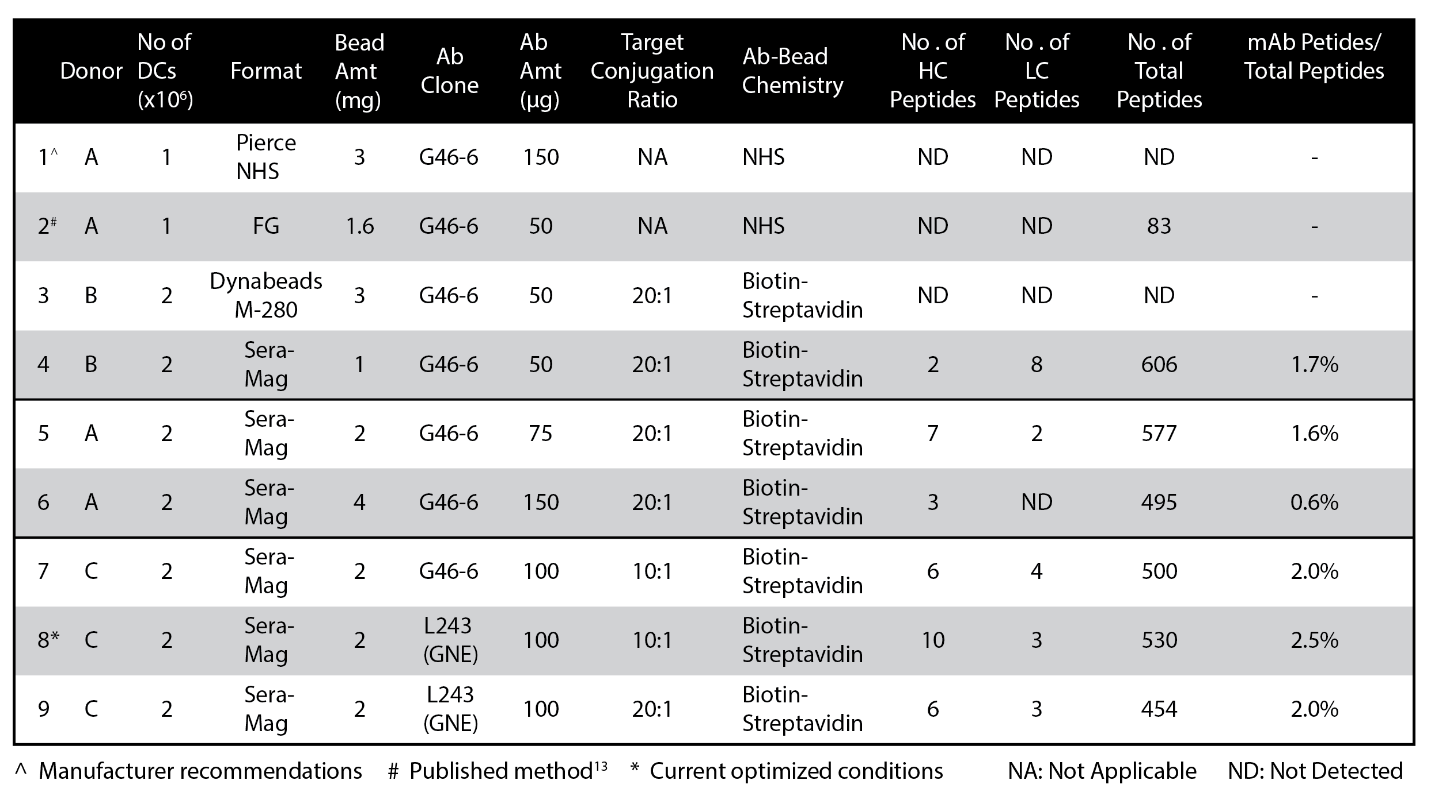
**

**Supplemental Figures
Supplemental Figure 1.** Intact LC-MS analysis assessing biotin conjugation incorporation ratio (BIR) (**A**) A 10:1 conjugation ratio (CR) was sufficient for biotin conjugation of aHLA-DR Ab G46-6. (**B**) Biotin incorporation of aHLA-DR L243 at a 10:1 or 20:1 CR is greater than that for G46-6.


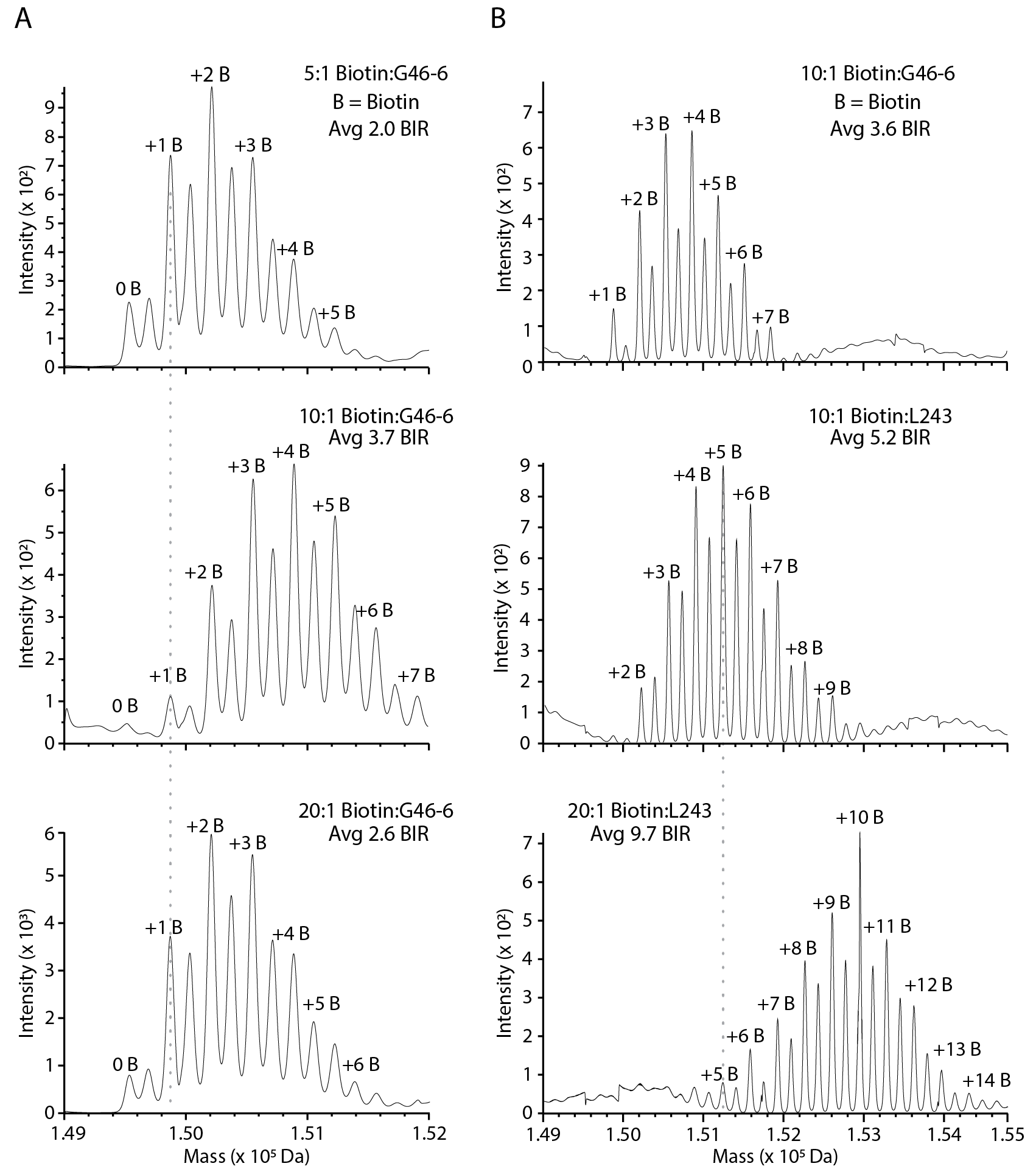


**Supplemental Figure 2.**  Heat map comparison of mAb A peptides detected using Tonobo Biotech L243 or GNE L243 as compared to G46-6. (**A**) BD G46-6 and Tonbo L243 aHLA-DR mAbs perform comparably in IA capture as assessed by peptide and cluster detection. (**B**) BD G46-6 and GNE in-house L243 aHLA-DR mAbs perform comparably in IA capture as assessed by peptide and cluster detection. The 10:1 and 20:1 biotin CR mAbs identify the same peptides and clusters; thus a 10:1 CR was deemed sufficient.

**
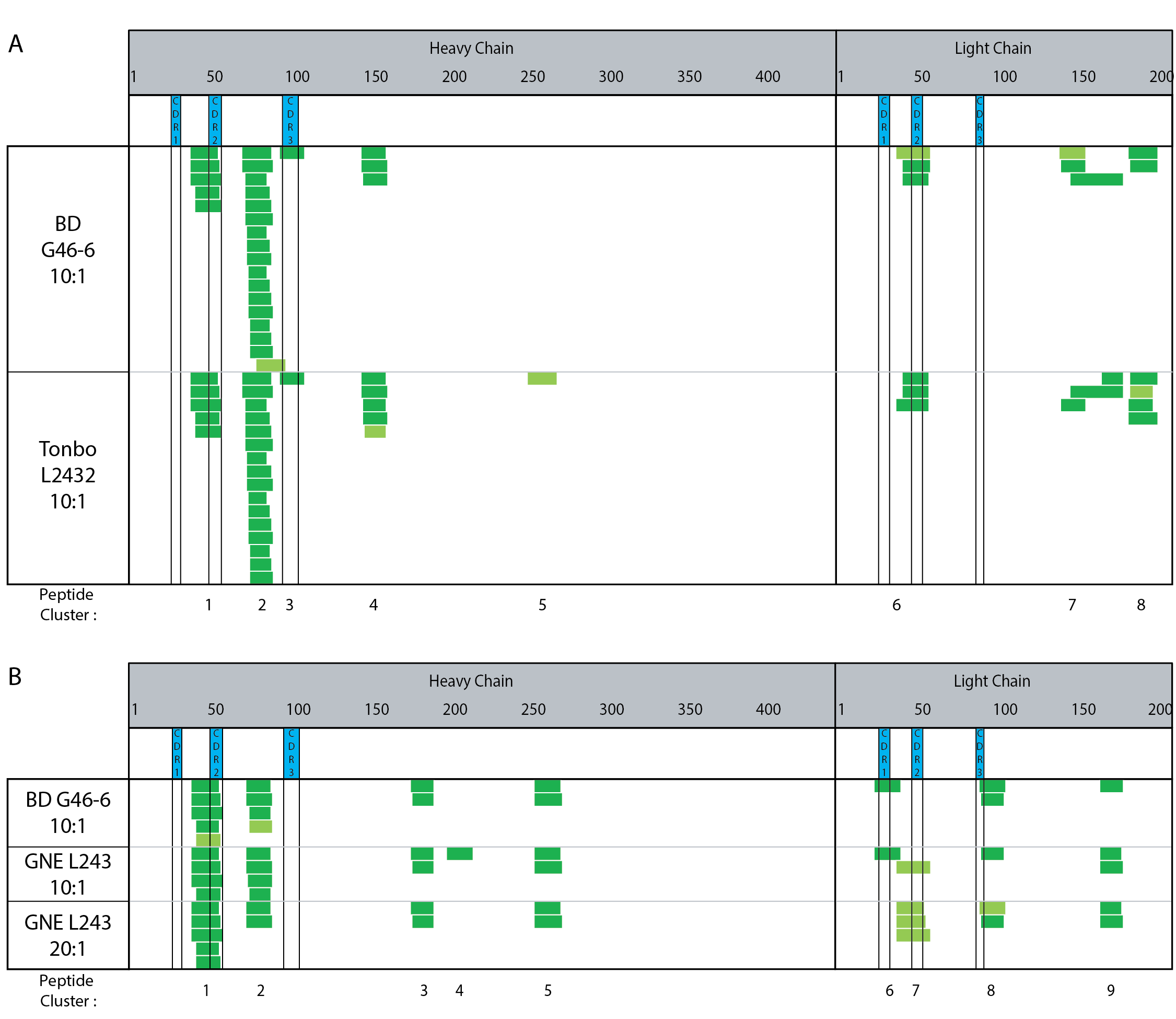
**

**Supplemental Figure 3.** Distribution of peptide lengths. A median peptide length of 16 amino acid residues is observed for (**A**) biotherapeutic peptides and (**B**) self, non-biotherapeutic peptides


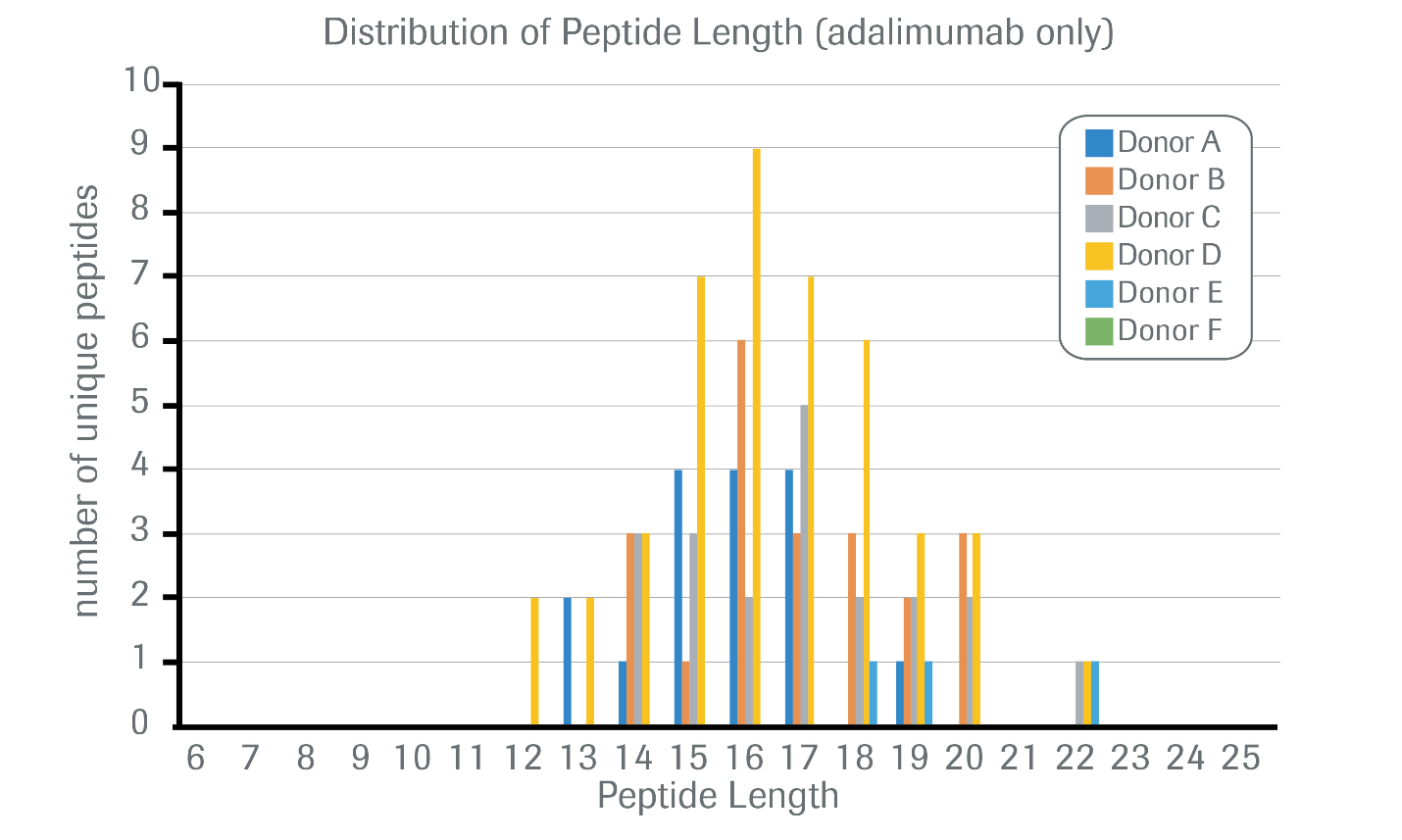


A

**
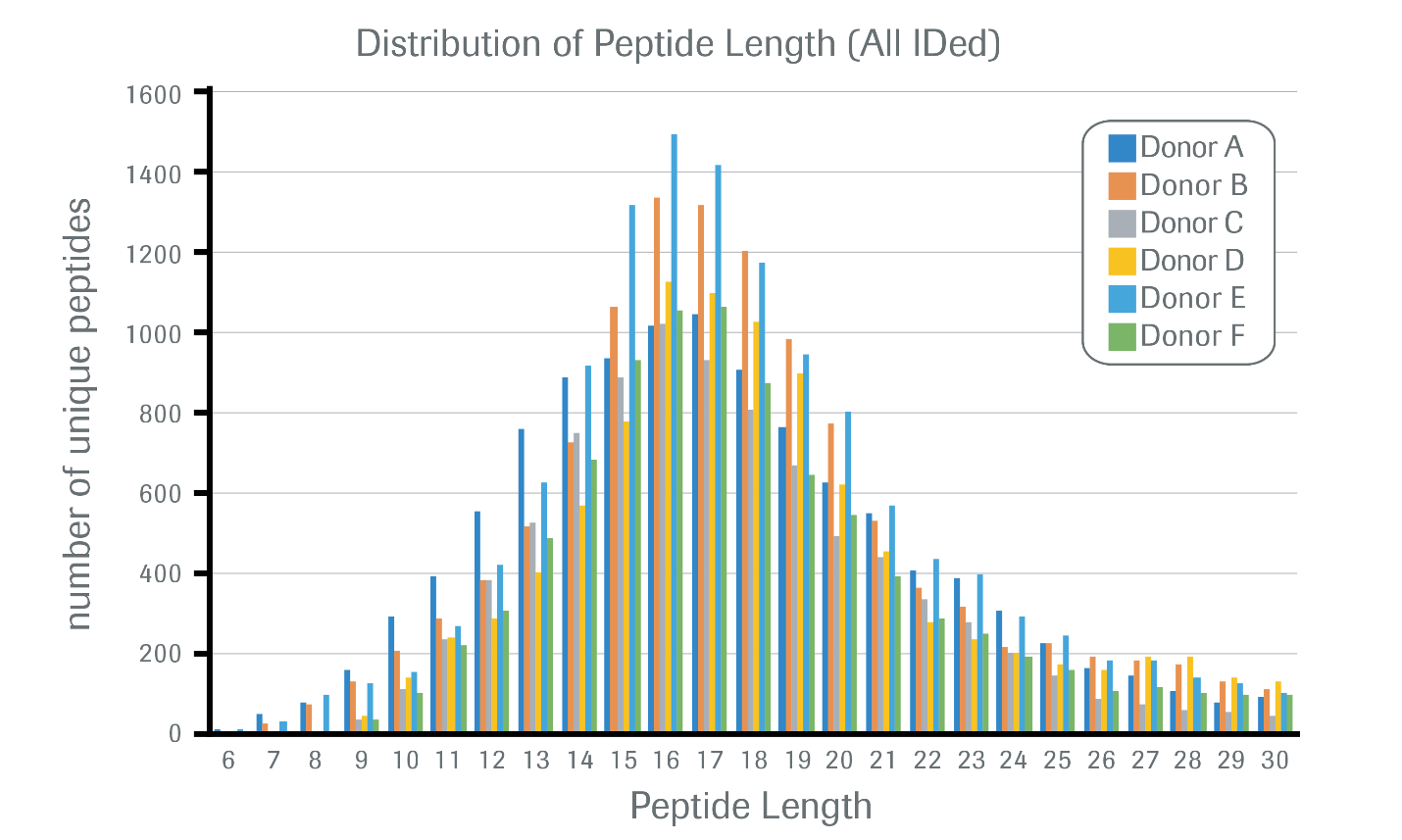
**

B

**Supplemental Figure 4.** Comparison of clinical reported ADA versus number observed clusters or number of peptides (normalized per preparation).


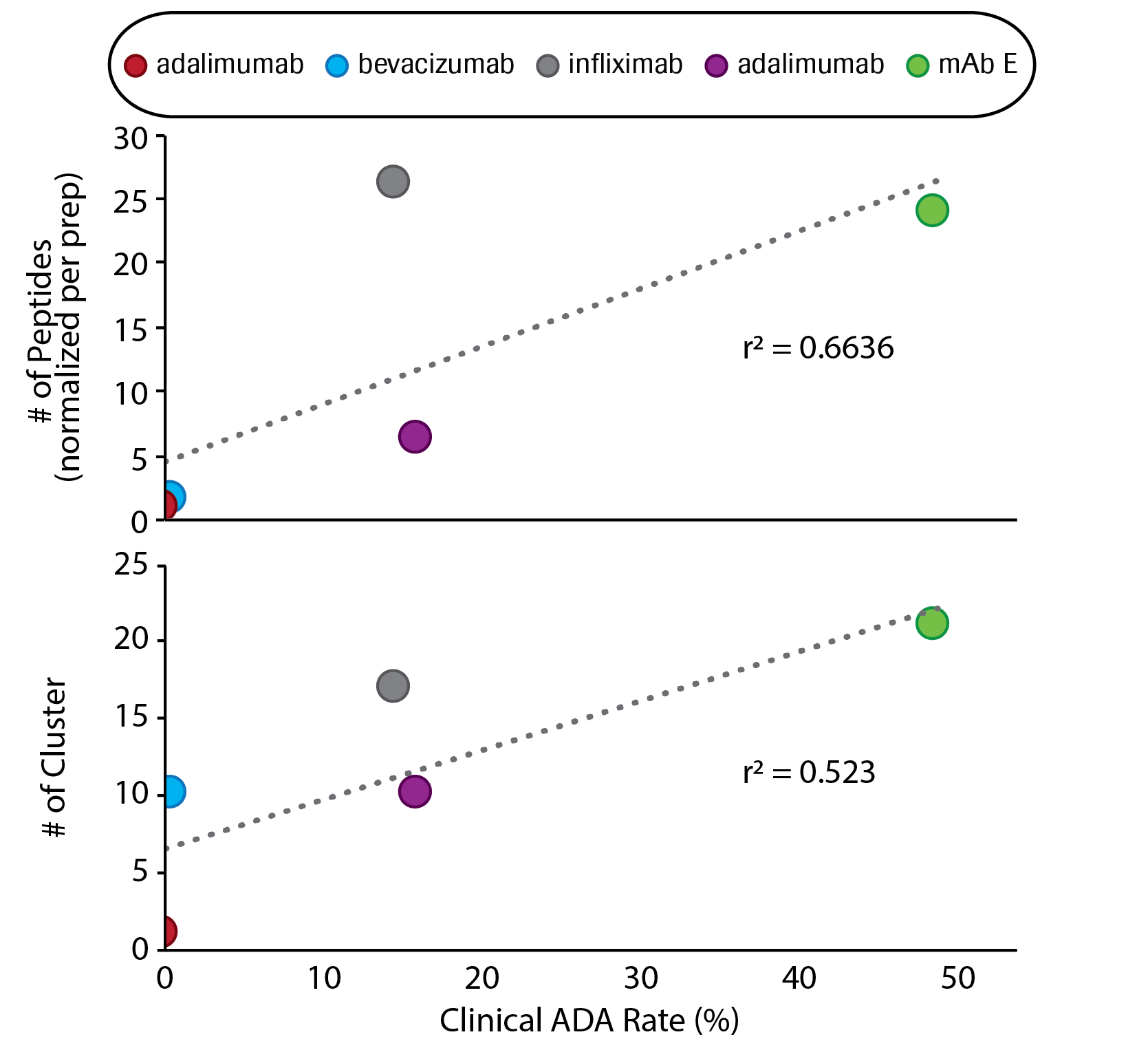


**Supplemental Methods
Supplemental Method 1.** KingFisher Method for preparation of beads to be used in HLA-DR immunoaffinity capture.


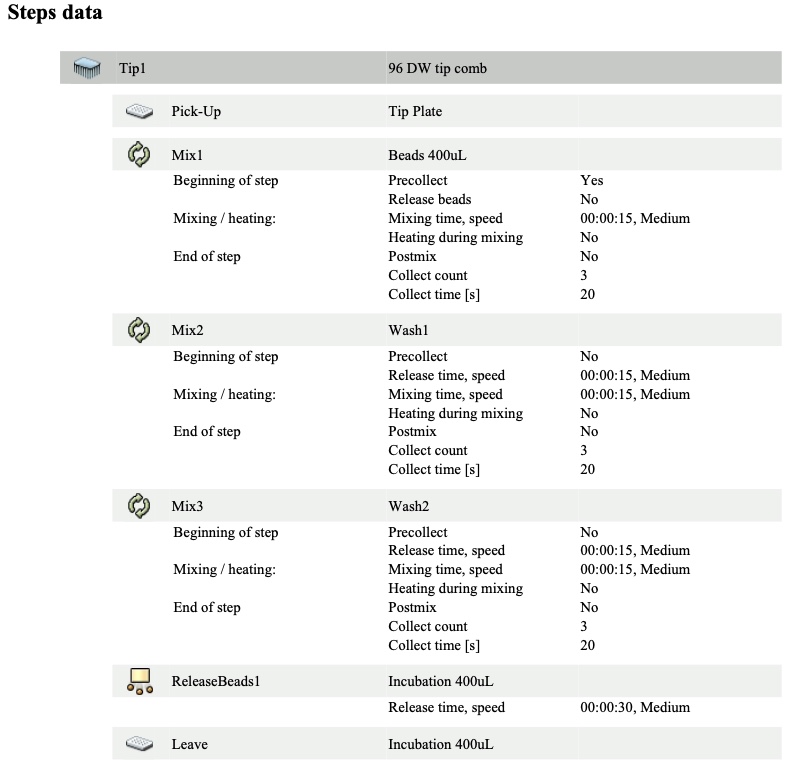


**Supplemental Method 2.** KingFisher method for washing beads post- HLA-DR immunoaffinity capture.


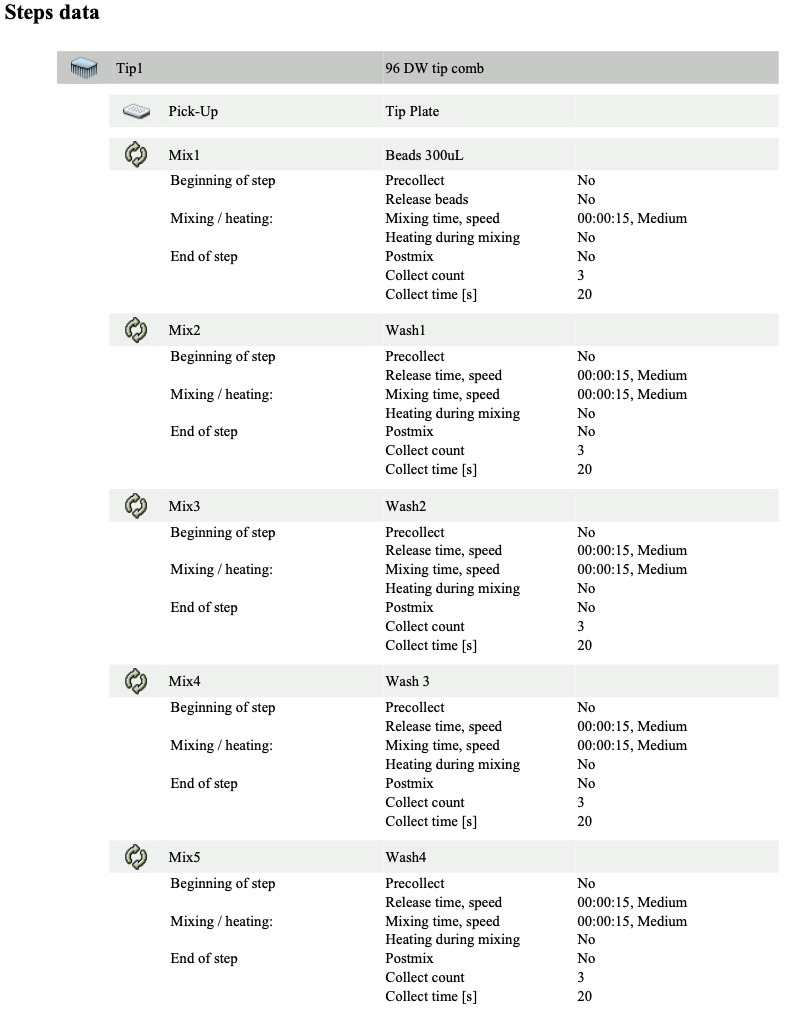


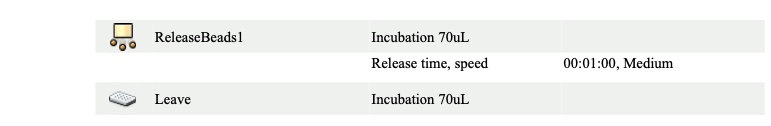


**Supplemental Method 3.** Computational Proteomic Data Analysis Utilizing Percolator. Percolator is used in place of Fixed Value PSM to minimize false-positive peptide identifications. All other settings were the same.


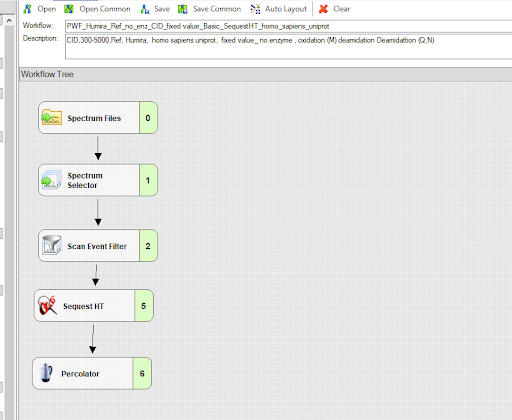


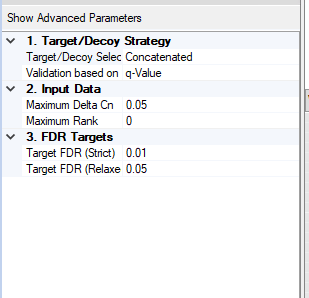

Supplement: Supplementary file 1 [file DataSheet_1.docx]
